# Author Correction: DrugnomeAI is an ensemble machine-learning framework for predicting druggability of candidate drug targets

**DOI:** 10.1038/s42003-023-05086-5

**Published:** 2023-07-11

**Authors:** Arwa Raies, Ewa Tulodziecka, James Stainer, Lawrence Middleton, Ryan S. Dhindsa, Pamela Hill, Ola Engkvist, Andrew R. Harper, Slavé Petrovski, Dimitrios Vitsios

**Affiliations:** 1 Centre for Genomics Research, Discovery Sciences, BioPharmaceuticals R&D, AstraZeneca, Cambridge, UK; 2 Centre for Genomics Research, Discovery Sciences, BioPharmaceuticals R&D, AstraZeneca, Waltham, MA USA; 3 Department of Molecular and Human Genetics, Baylor College of Medicine, Houston, USA; 4 Emerging Innovations, Discovery Sciences, BioPharmaceuticals R&D, AstraZeneca, Waltham, MA USA; 5 Molecular AI, Discovery Sciences, R&D, AstraZeneca, Gothenburg, Sweden; 6 Department of Medicine, University of Melbourne, Austin Health, Melbourne, VIC Australia

**Keywords:** Machine learning, Target identification, Bioinformatics, Computational models

Correction to: *Communications Biology* 10.1038/s42003-022-04245-4, published online 24 November 2022.

In this article the hyperlink provided in the Code Availability statement in the sentence beginning ‘The DrugnomeAI package…’ was incorrect. The original article has been corrected.

