# Supplementary material for: DrugnomeAI is an ensemble machine-learning framework for predicting druggability of candidate drug targets
Source: Commun Biol. 2022 Nov 24;5:1291. doi: 10.1038/s42003-022-04245-4 (PMC9700683; doi:10.1038/s42003-022-04245-4)
Supplement: Supplementary file 2 — Supplementary Information [file 42003_2022_4245_MOESM2_ESM.pdf]

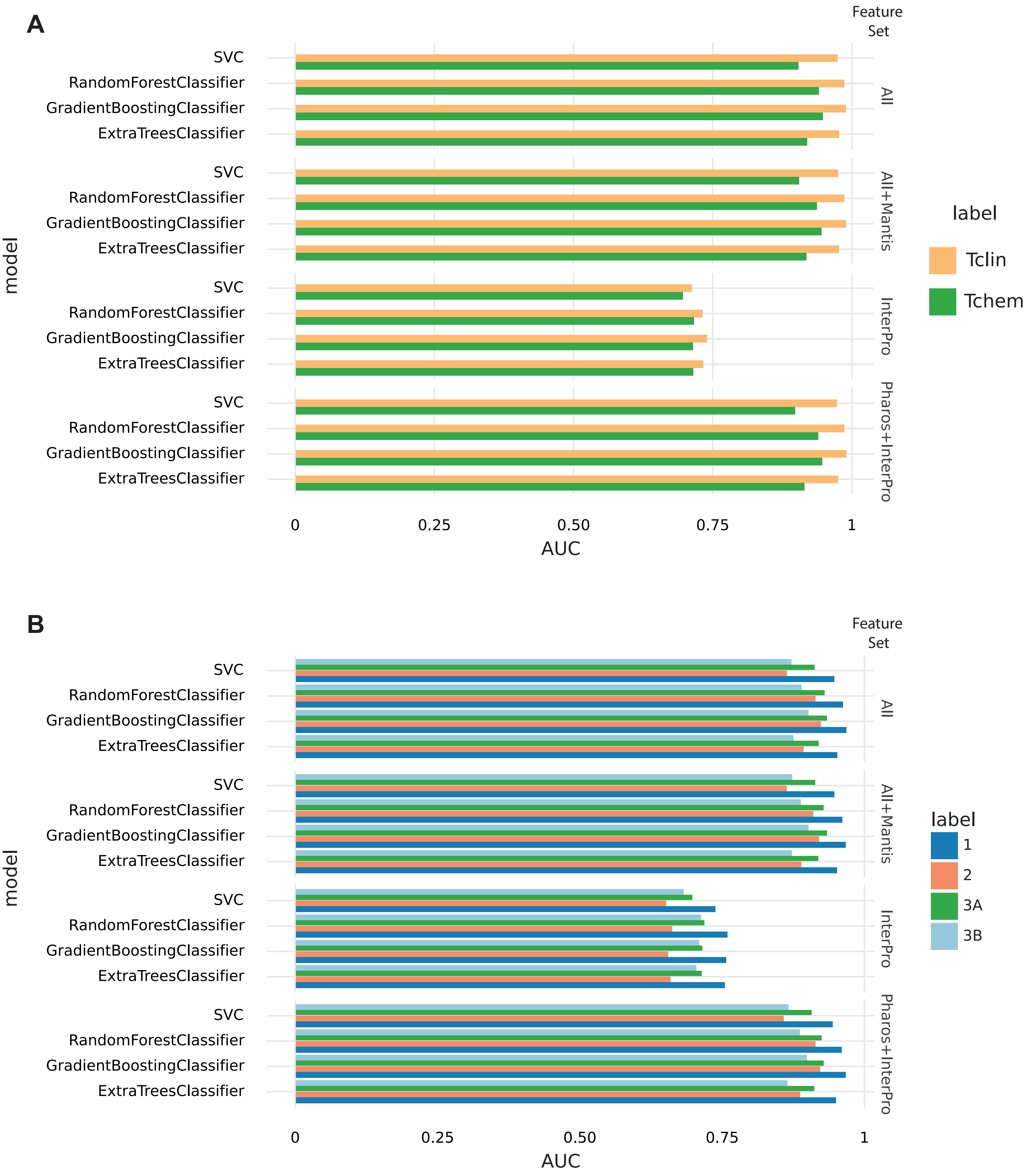

Supplementary Fig. 1 Detailed breakdown of AUC score results yielded for all the classifiers across the considered feature sets for (A) Pharos labels. (B) Triage labels.

A

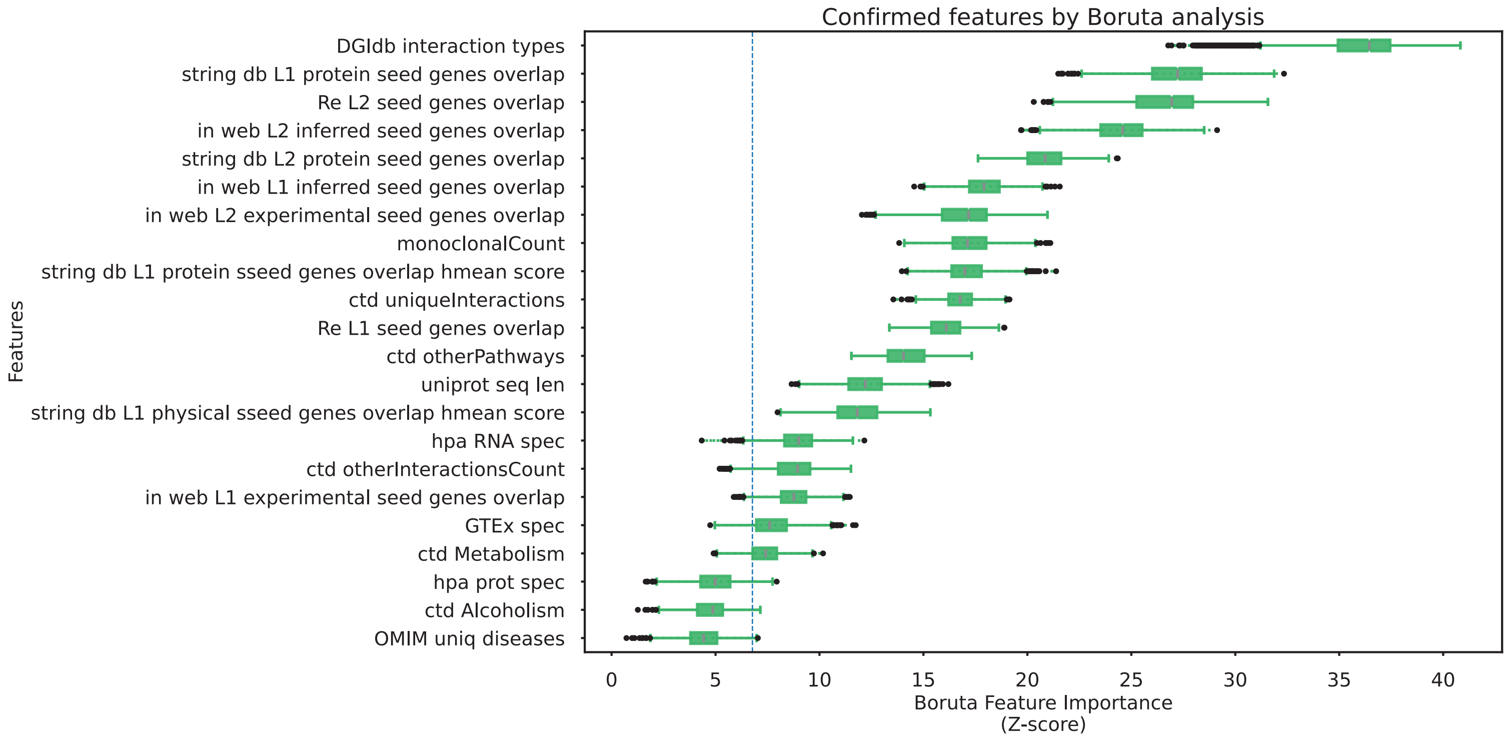

B

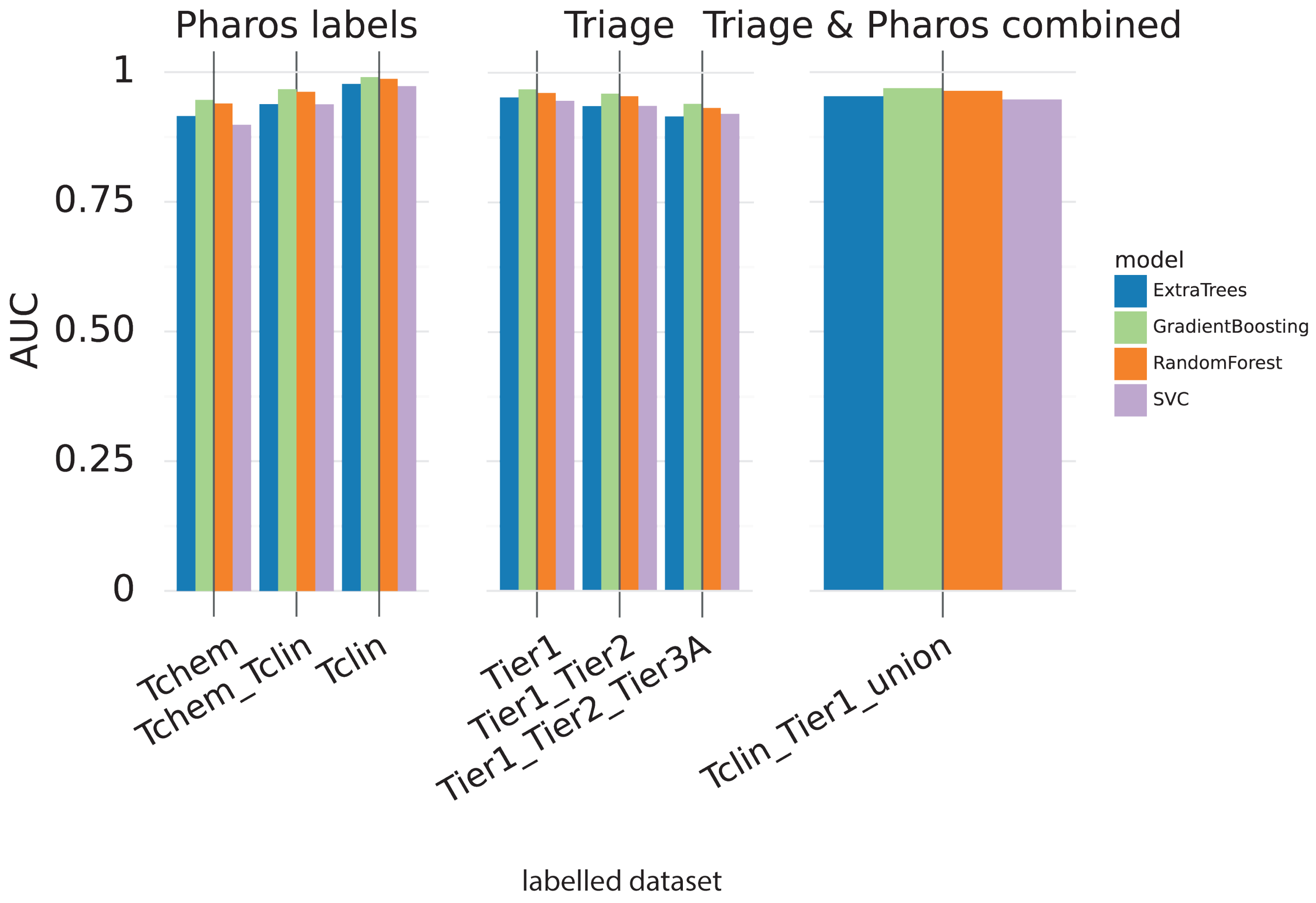

Supplementary Fig. 2 A) List of the relevant features confirmed by Boruta algorithm for dataset labelled with Tier 1. B) Comparison of the classifiers' AUC score results across different labelling variants utilizing the druggability-specific dataset

A

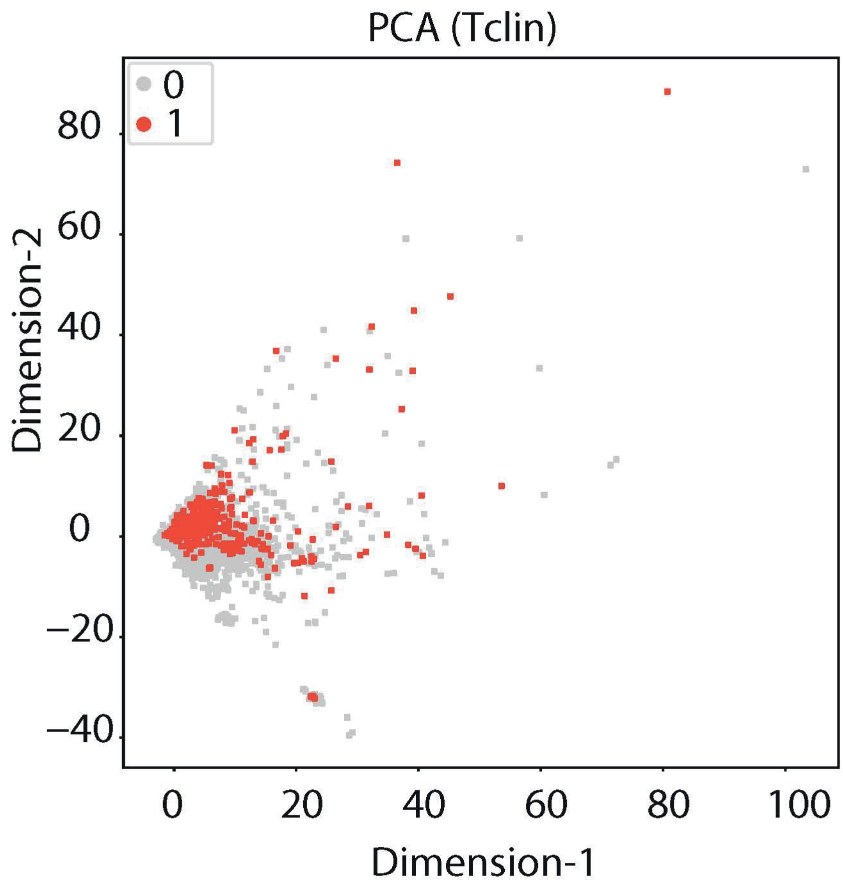

B

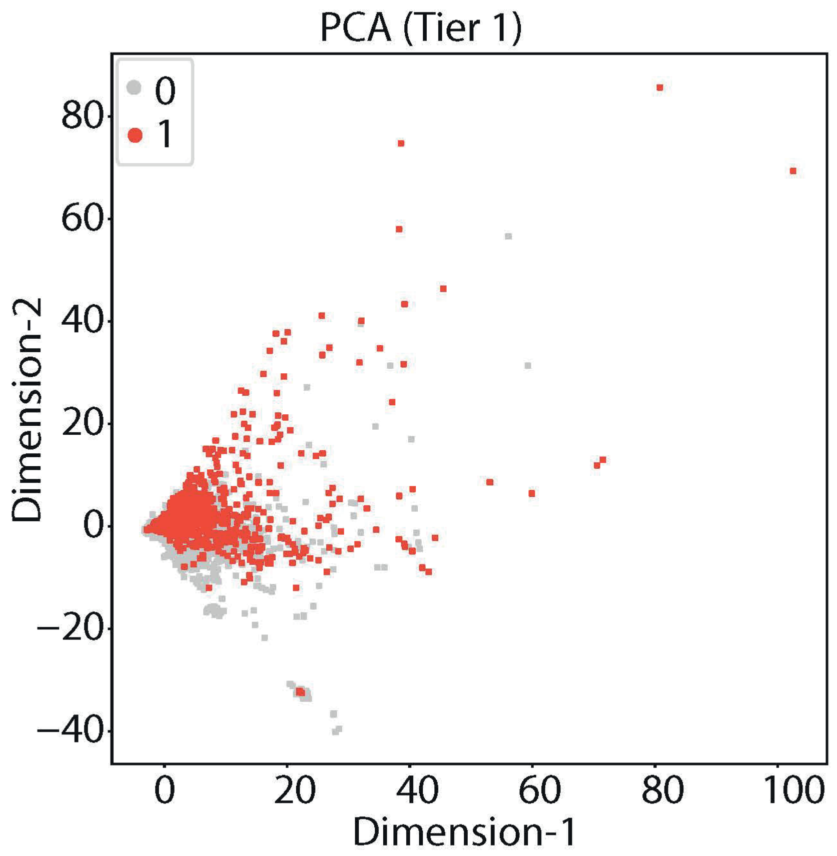

Supplementary Fig. 3 Principal component analysis (PCA) of gene features for (A) Tclin and (B) Tier 1 datasets. Red circles indicate known targets.

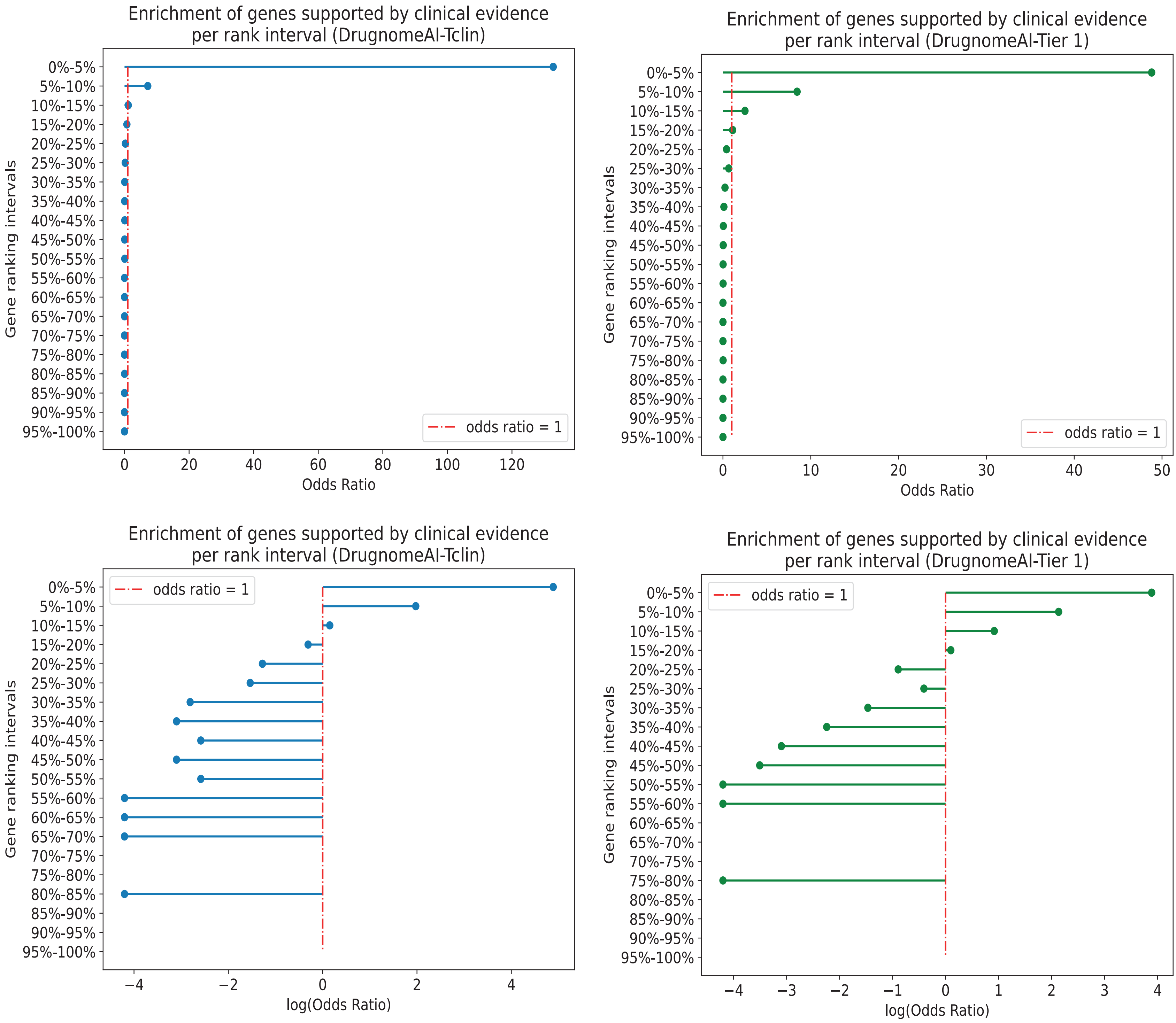

Supplementary Fig. 4 Enrichment of top 5% ranked gene by DrugnomeAI among genes with clinical evidence (odds ratio). 0%-5% indicates genes ranked in the top 5% while 95%-10-0% indicates the lowest ranked genes.

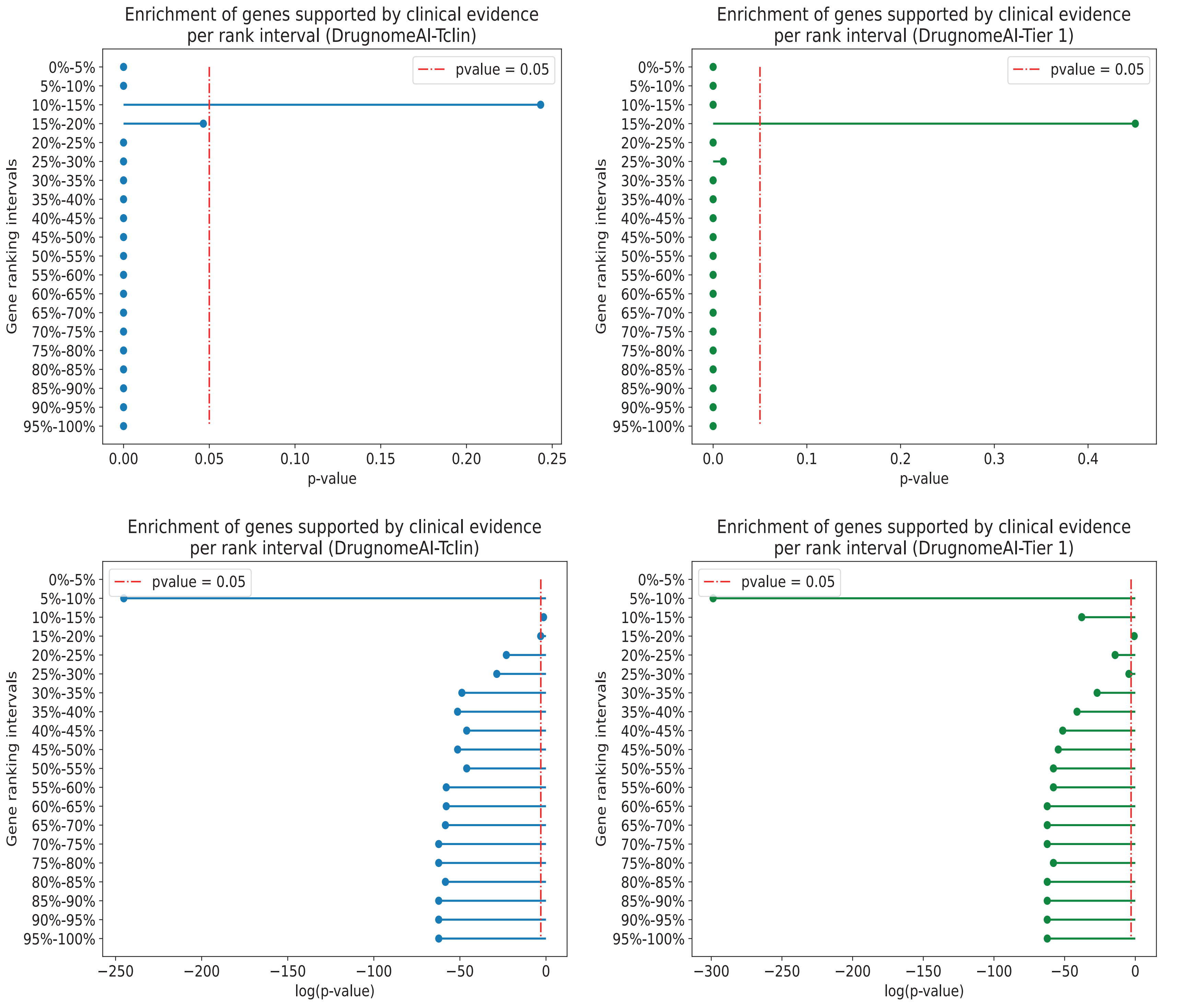

Supplementary Fig. 5 Enrichment of top 5% ranked genes by DrugnomeAI among genes with clinical evidence. 0%-5% indicates genes ranked in the top 5% while 95%-10-0% indicates the lowest ranked genes.

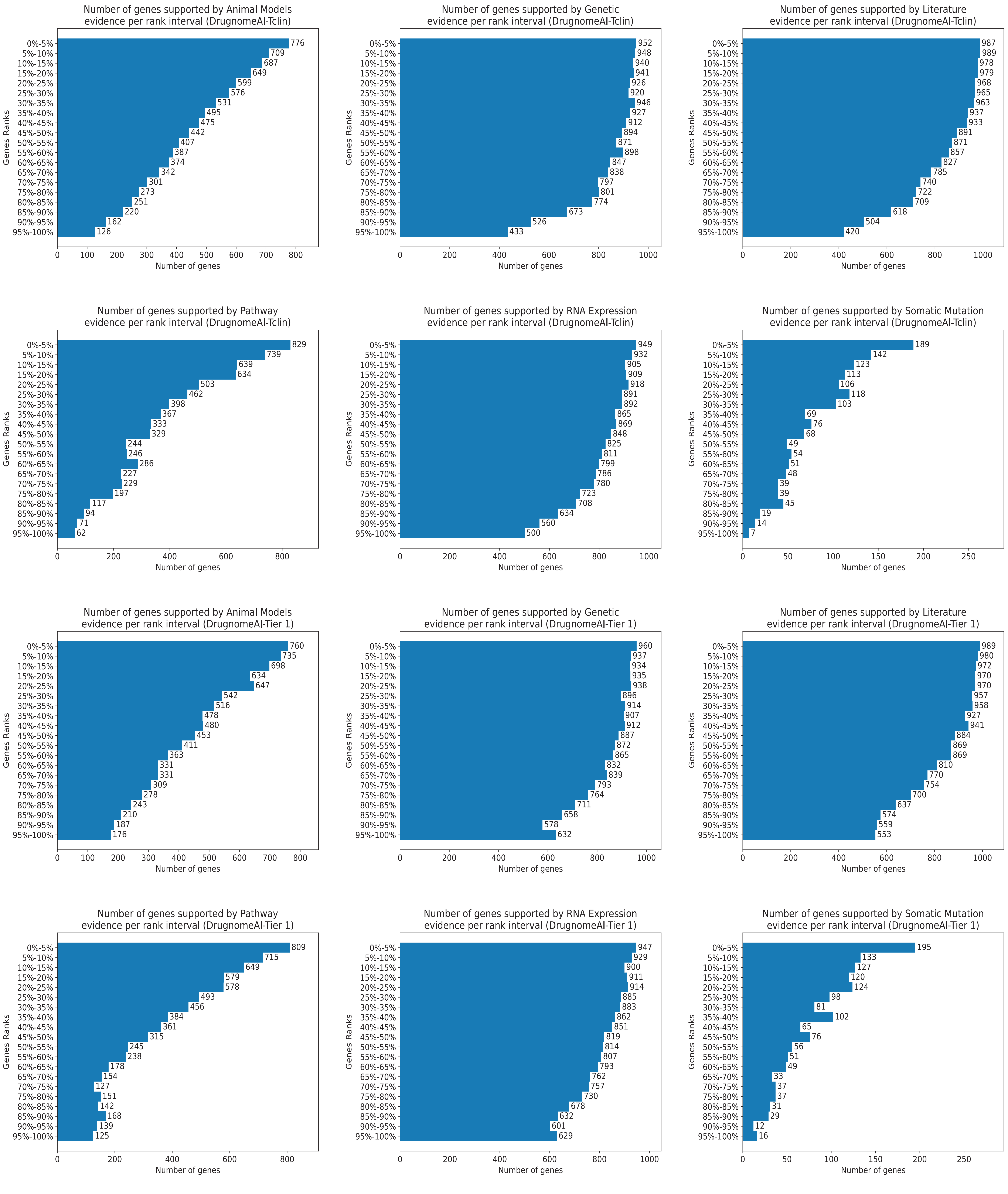

Supplementary Fig. 6 Number of genes in the top 5% ranked by DrugnomeAI supported by each evidence type (non-clinical evidence). 0%-5% indicates genes ranked in the top 5% while 95%-10-0% indicates the lowest ranked genes.

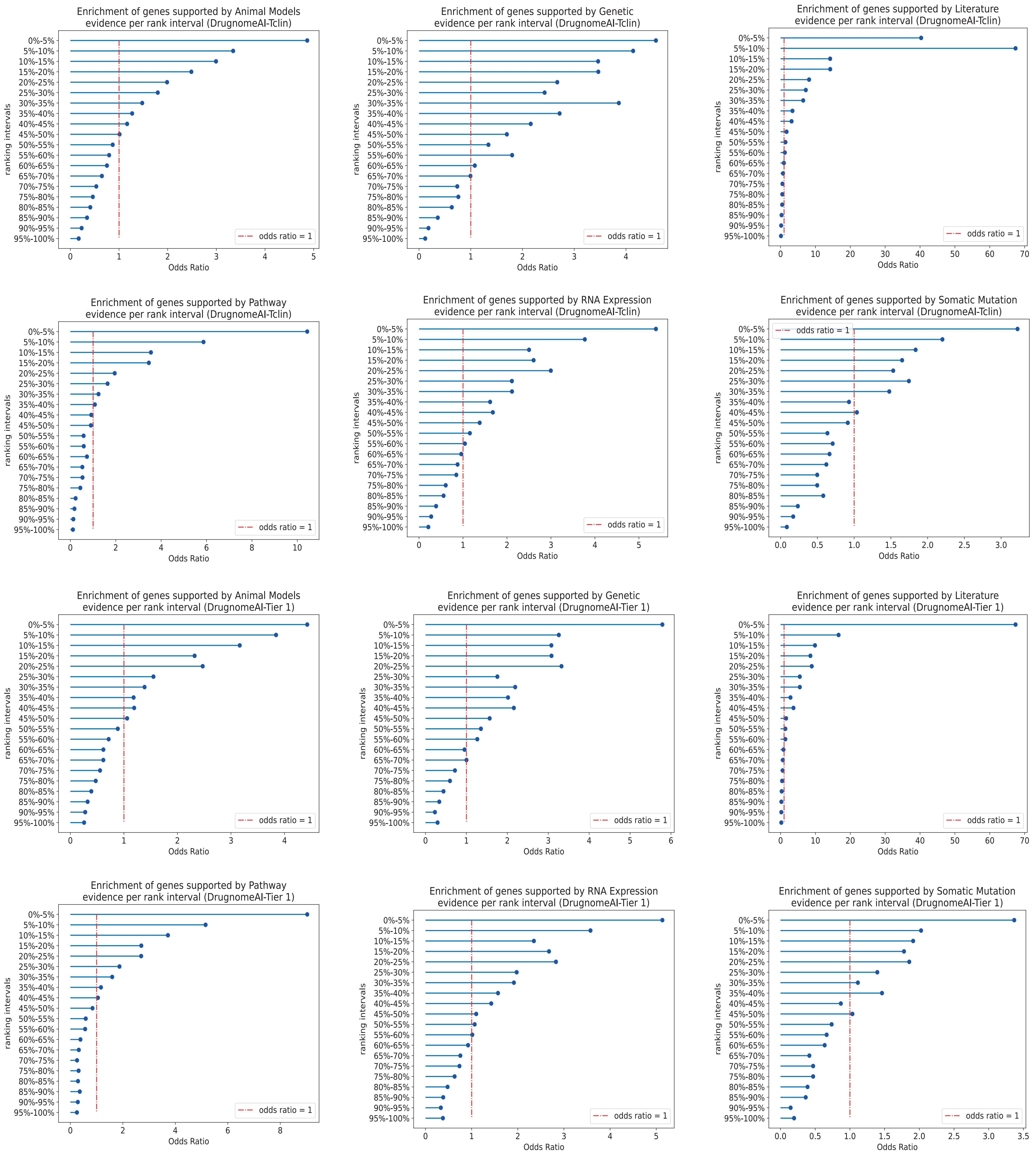

Supplementary Fig. 7 Enrichment of top 5% ranked genes by DrugnomeAI among genes supported by each evidence type (non-clinical evidence). 0%-5% indicates genes ranked in the top 5% while 95%-10-0% indicates the lowest ranked genes.

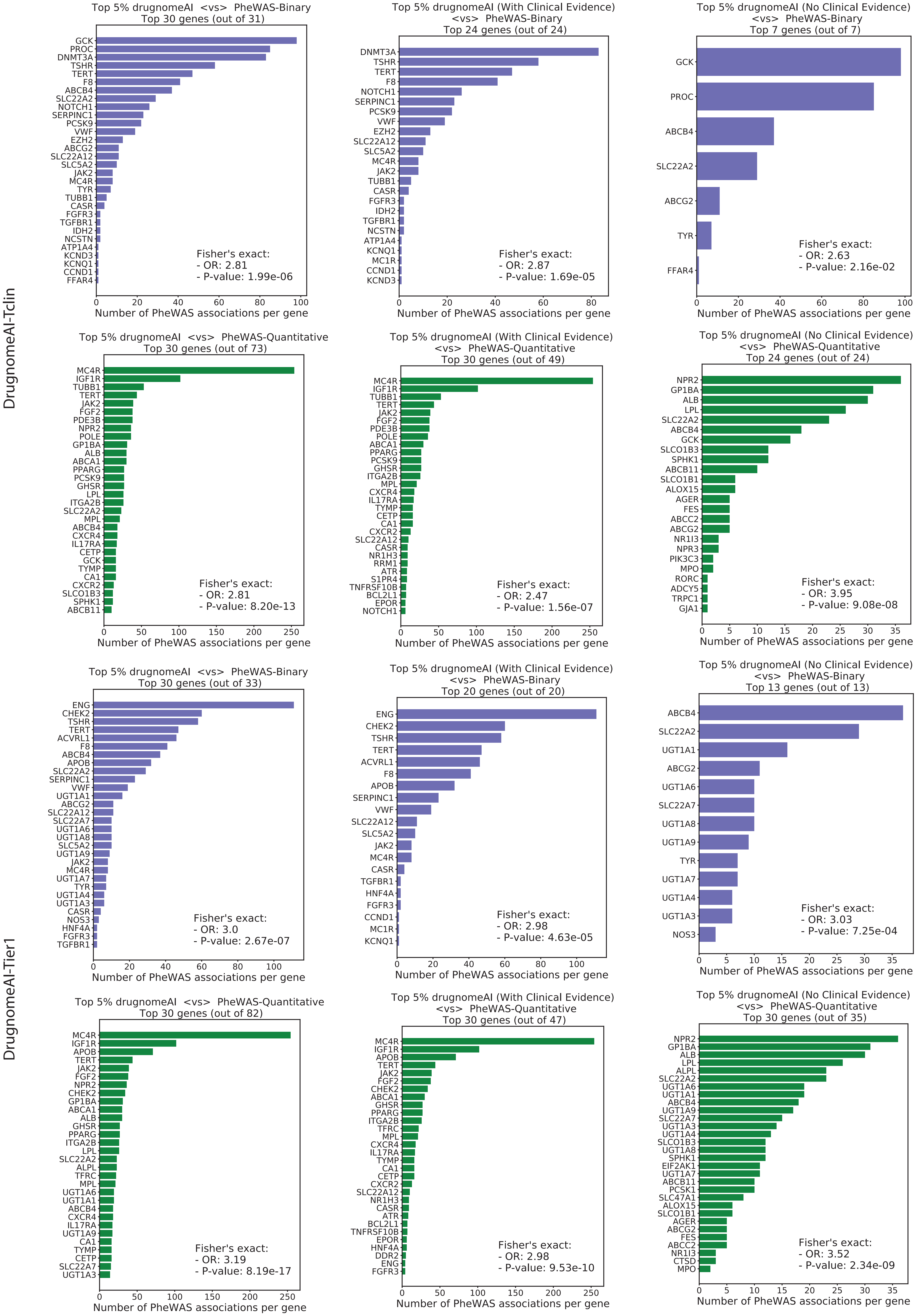

Supplementary Fig. 8 Enrichment of top 5% ranked genes by DrugnomeAI-Tclin and DrugnomeAI-Tier 1 among PheWAS top genes for binary and quantitative traits.

A

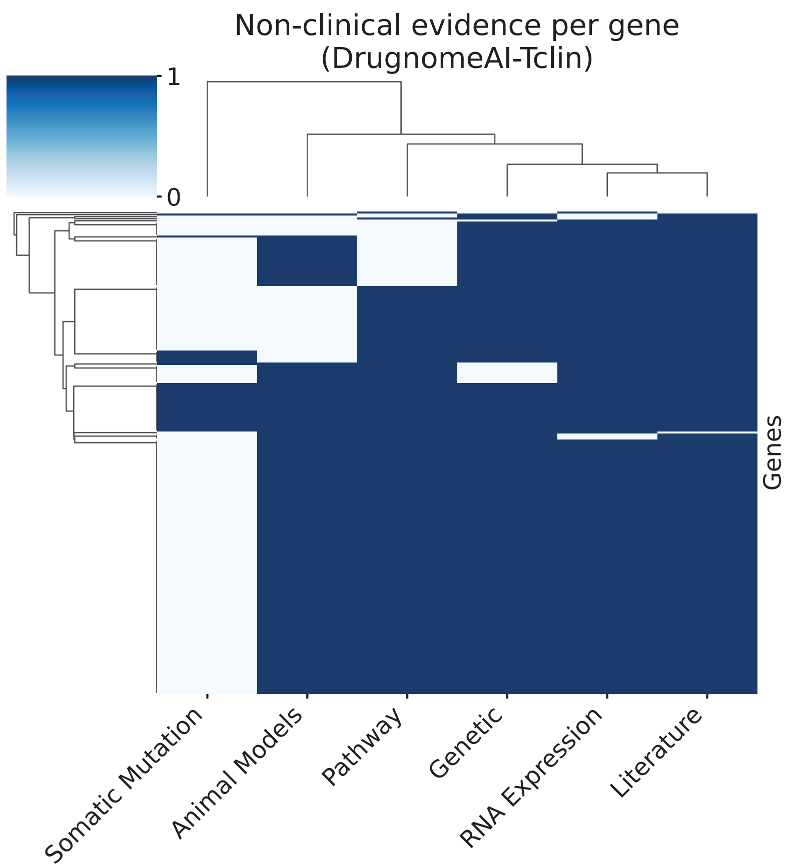

B

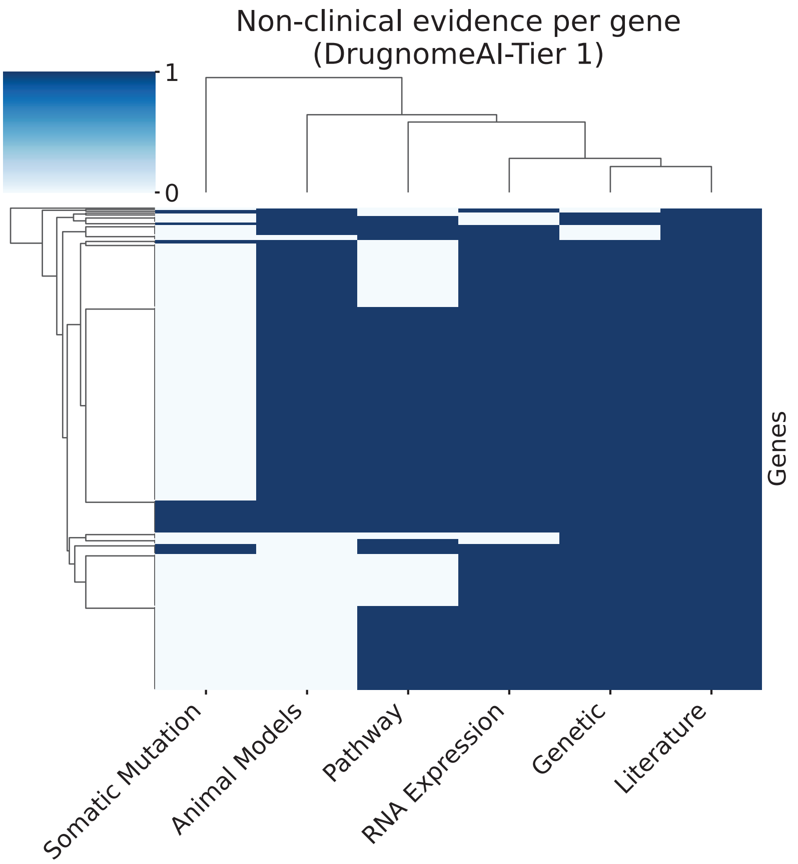

Supplementary Fig. 9 Non-clinical evidence of genes without clinical evidence in (A) Set1 and (B) Set2. Each row represents a gene, and columns correspond to evidence types. Each cell is a binary value indicating whether a gene is associated (blue cell) or not associated (white cell) with diseases with respect to each evidence type.

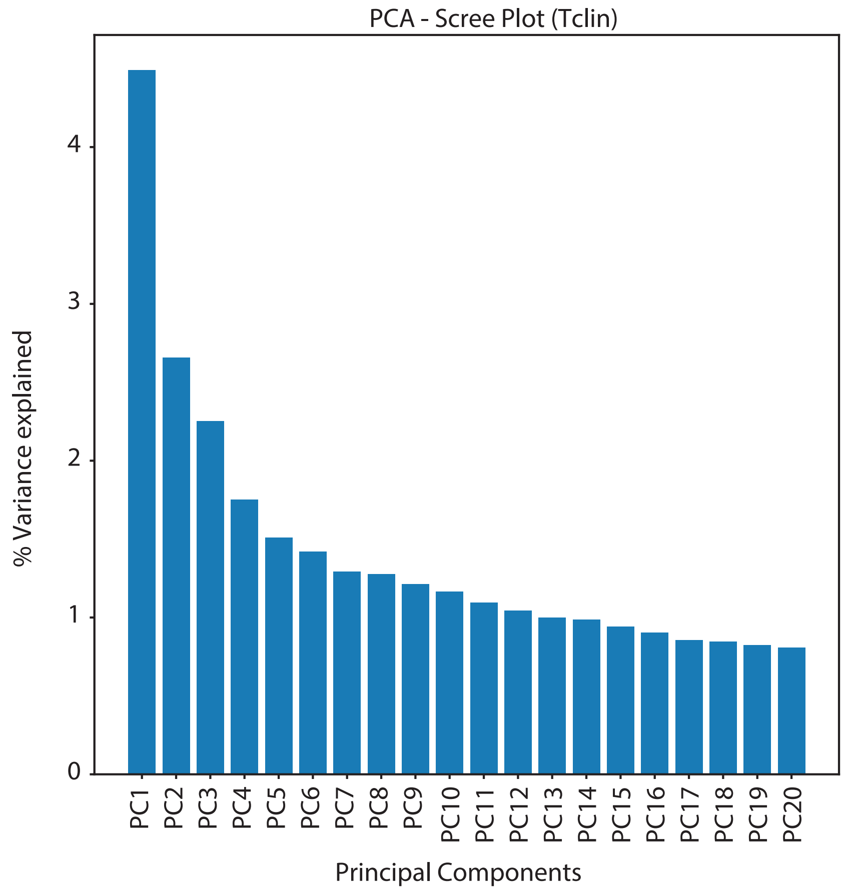

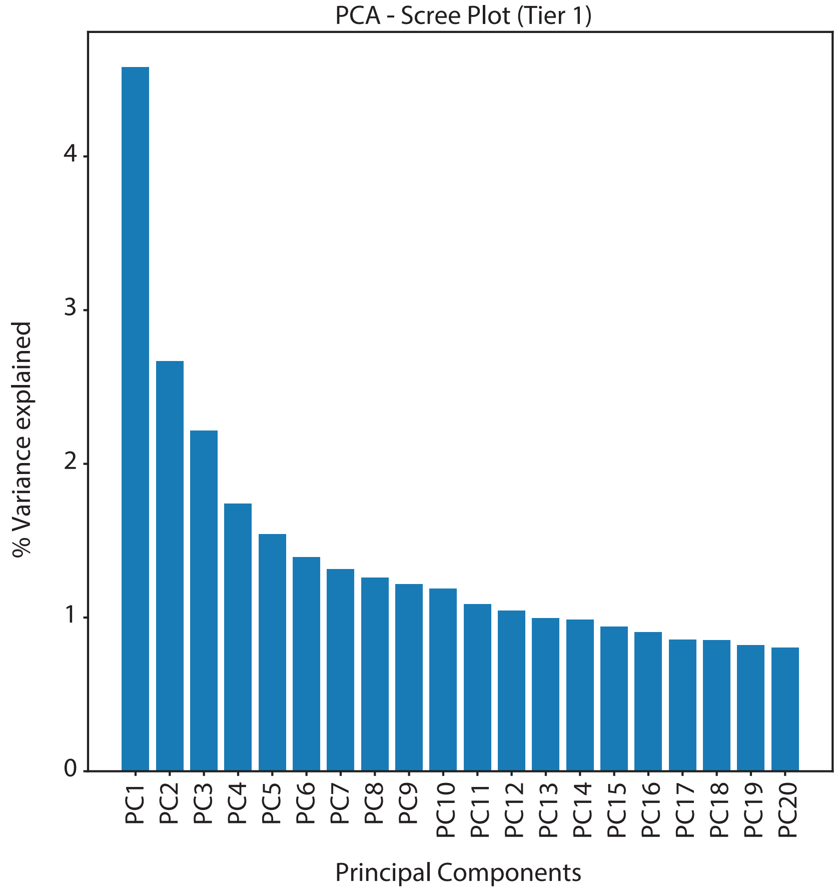

Supplementary Fig. 10 Variance explained by each principal component (PC) for Tclin and Tier 1 datasets.

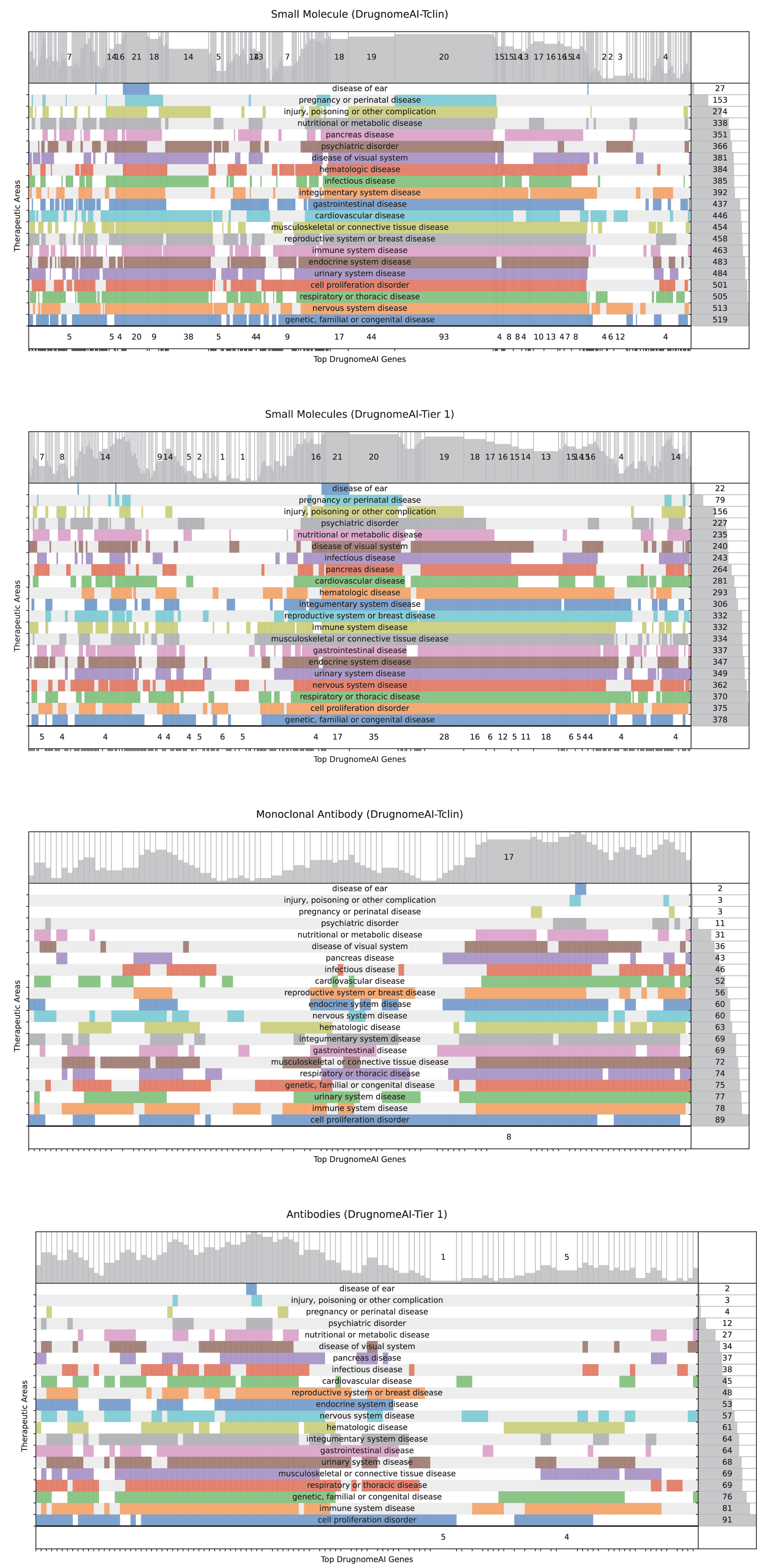

Supplementary Fig. 11. Therapeutic areas of top 5% ranked genes by DrugnomeAI. The bars on the right indicate the number of genes in top 5% genes in each therapeutic area. The bars on the top show the intersection between the theraeptuic areas (i.e., genes that belong to more than therapeutic areas).

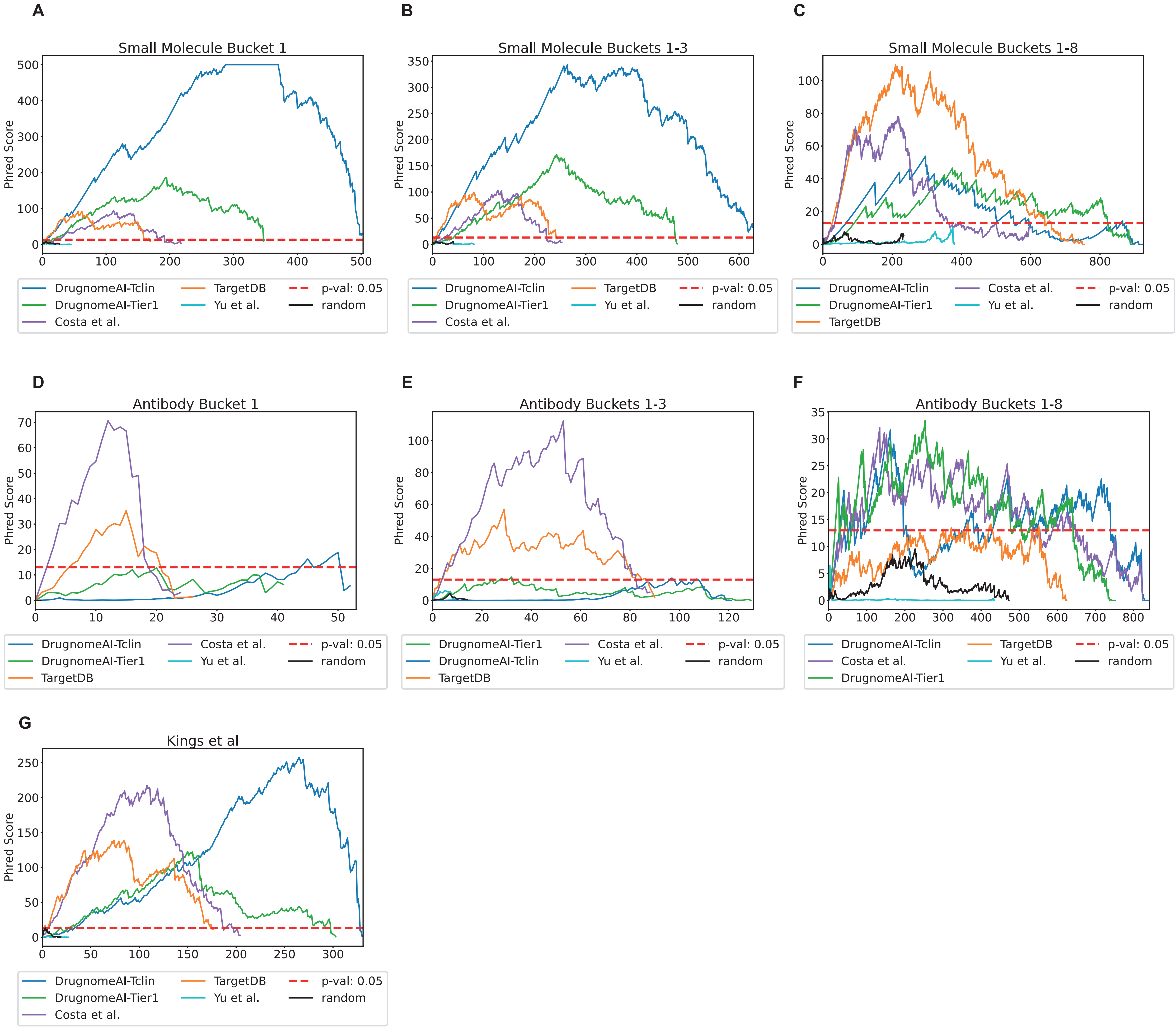

Supplementary Fig. 12 Enrichments of top 5% of genes ranked by DrugnomeAI-Tclin, DrugnomeAI-Tier1, Targes DB, and random model. The random model indicates genes are ranked randomly. The horizontal dashed red line represents the significant threshold of p-value = 0.05 for the hypergeometric tests. Phred values above the red line represent significant enrichment. The x-axis represents the overlap between top 5% predictions by each models and small molecule tractability ((A) Bucket 1, (B) Buckets 1-3, and (C) Buckets 1-8), antibody tractability data ((D) Bucket 1, (E)Buckets 1-3, and (F) Buckets 1-9), and genes with approved targets from King et all (G). The y-axis corresponds to the phred values from the hypergeometric tests. Larger phred values indicates higher significance.

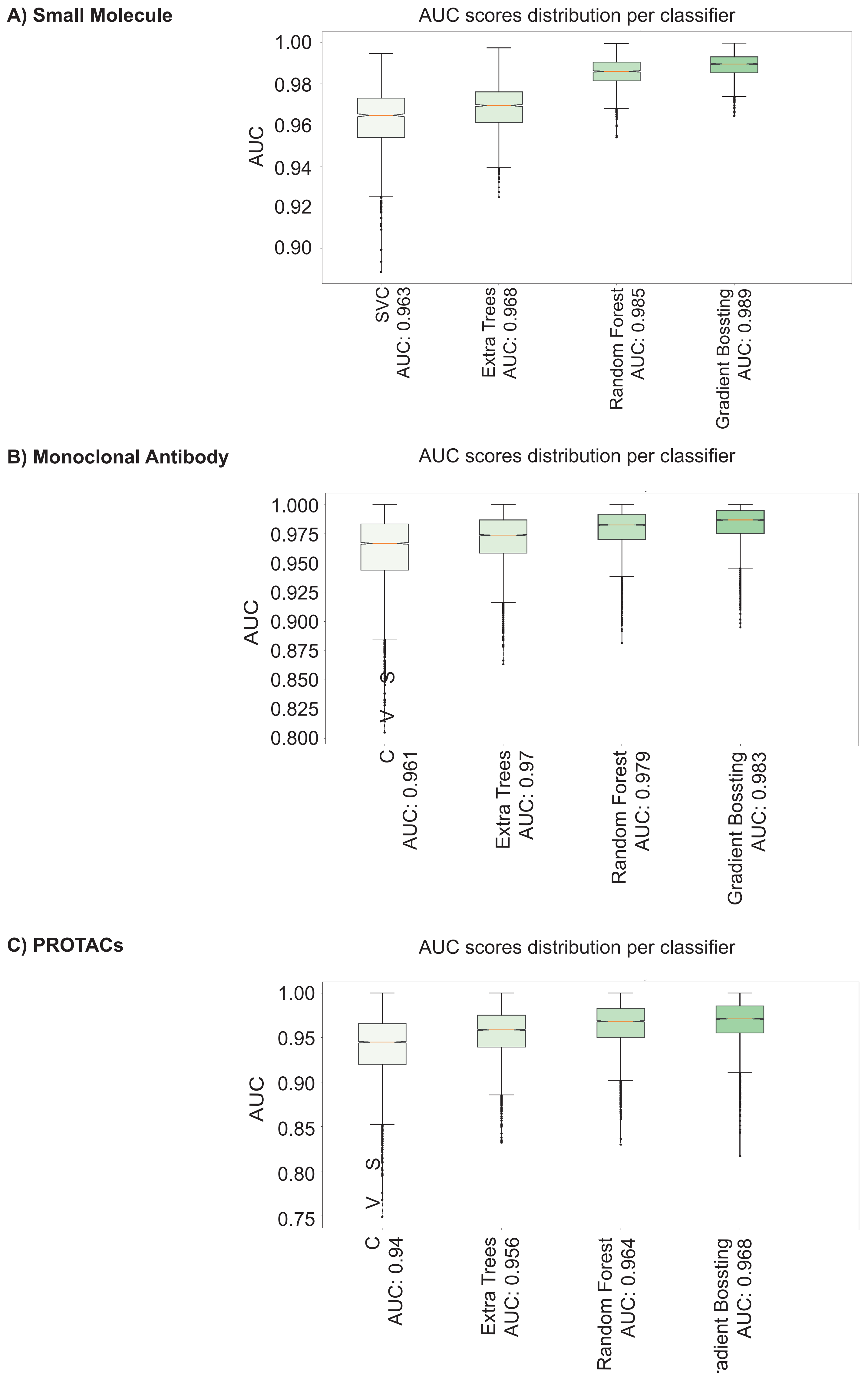

Supplementary Fig. 13 Model performance of therapeutic-modality models. The y-asis shows the AUC scores, and the x-axis corresponds to a classifier (SVC, Extra Trees, Random Forest, and Gradient Bossting).

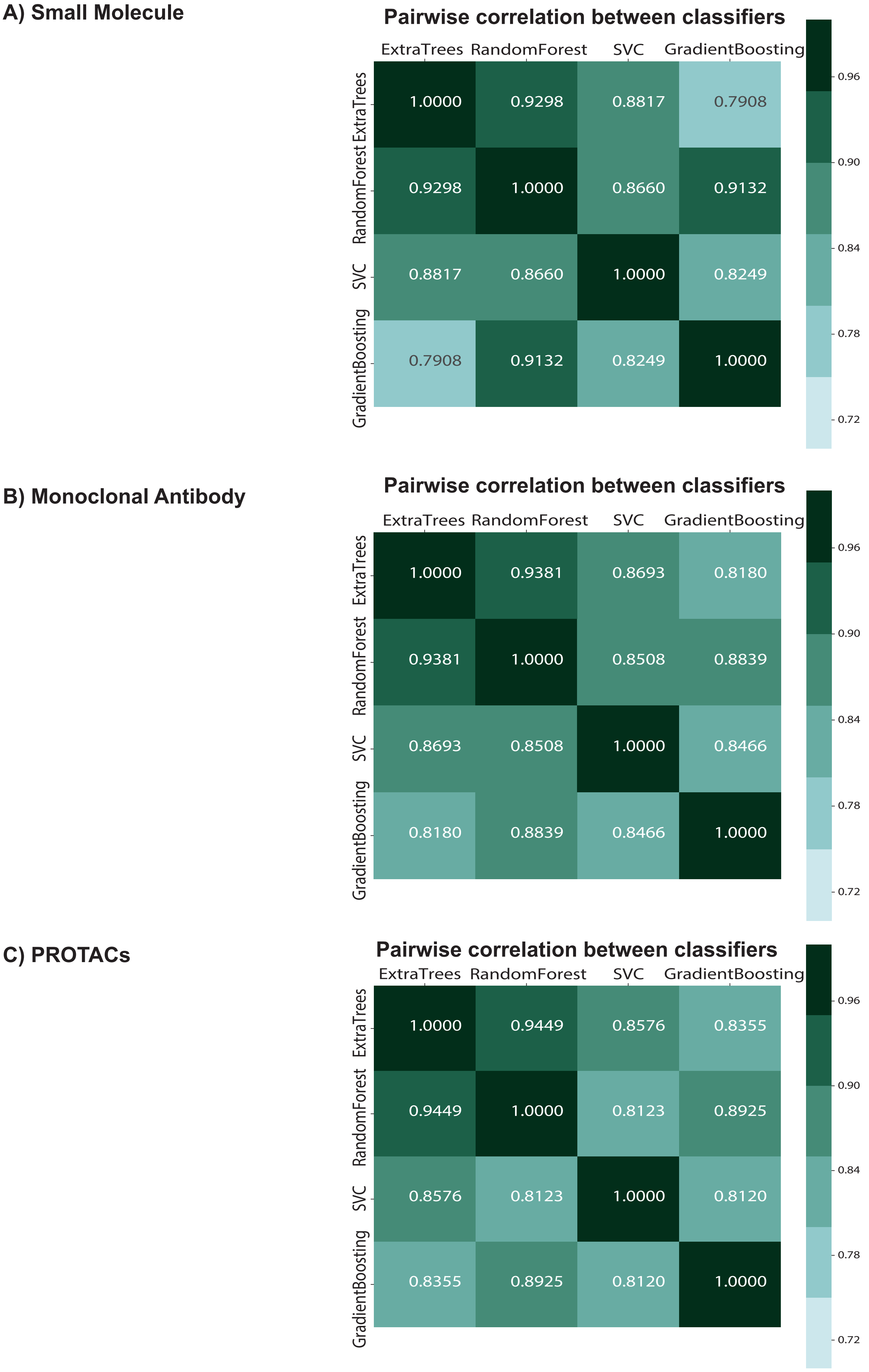

Supplementary Fig. 14 Correlations between four classifiers (Gradient Boosting, SVC, Random Forest, and Extra Trees) for each therapeutic modality. Higher scores indicate higher correlation.

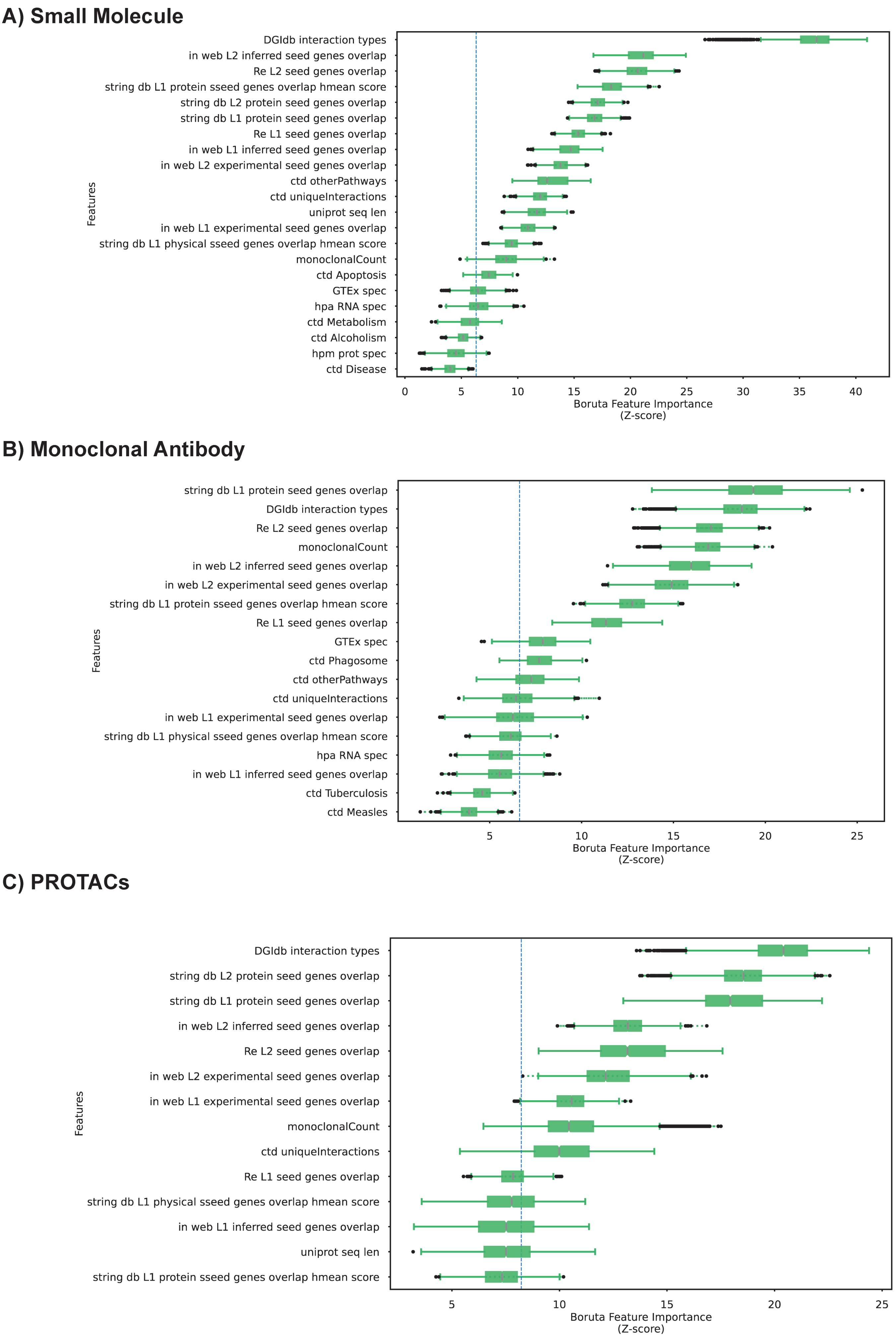

Supplementary Fig. 15 Confirmed features by Boruta analysis and novel genes in top 50 genes ranked per drug modality.

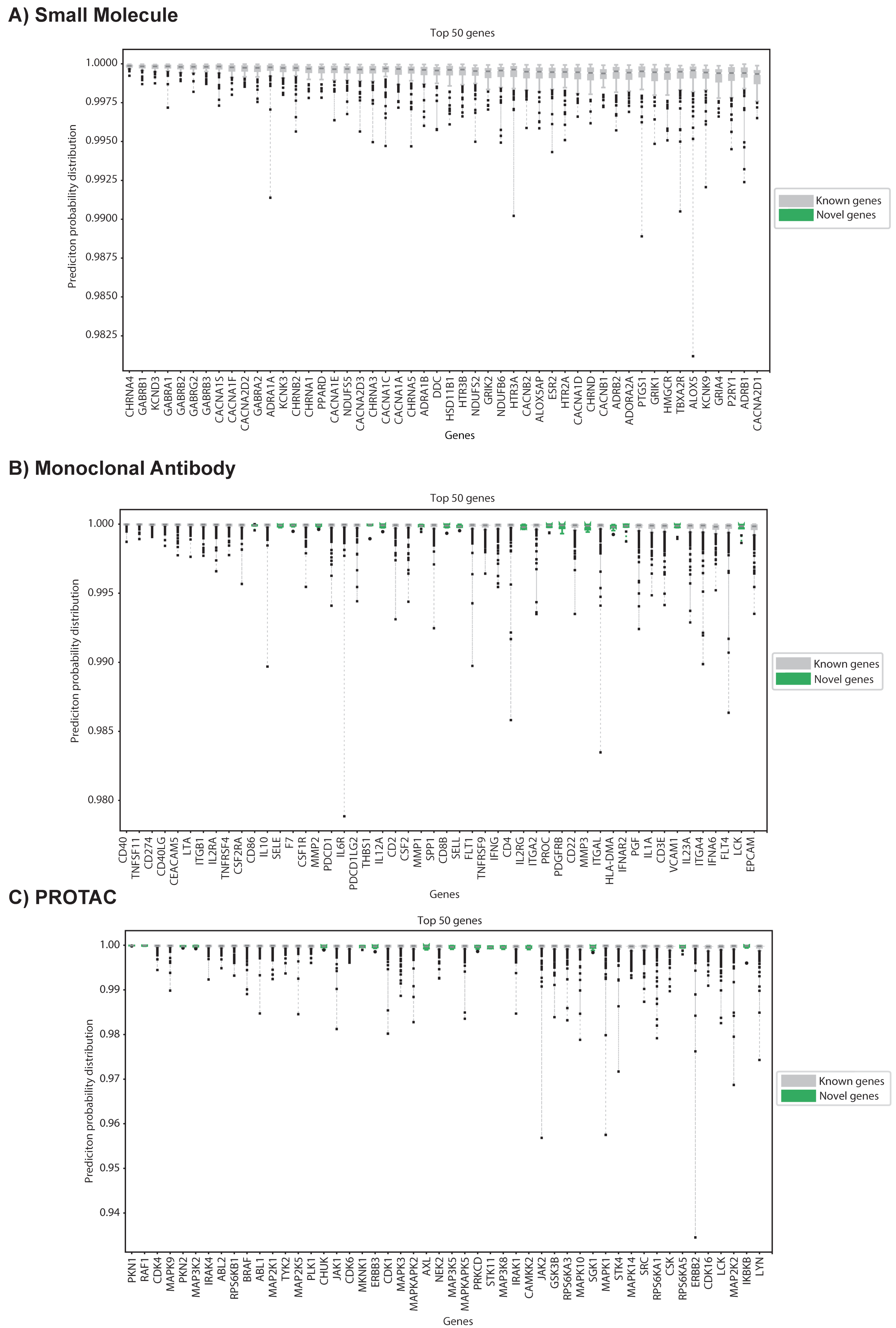

Supplementary Fig. 16 Novel genes in the top 50 genes by the gradient boosting model for each drug modality.

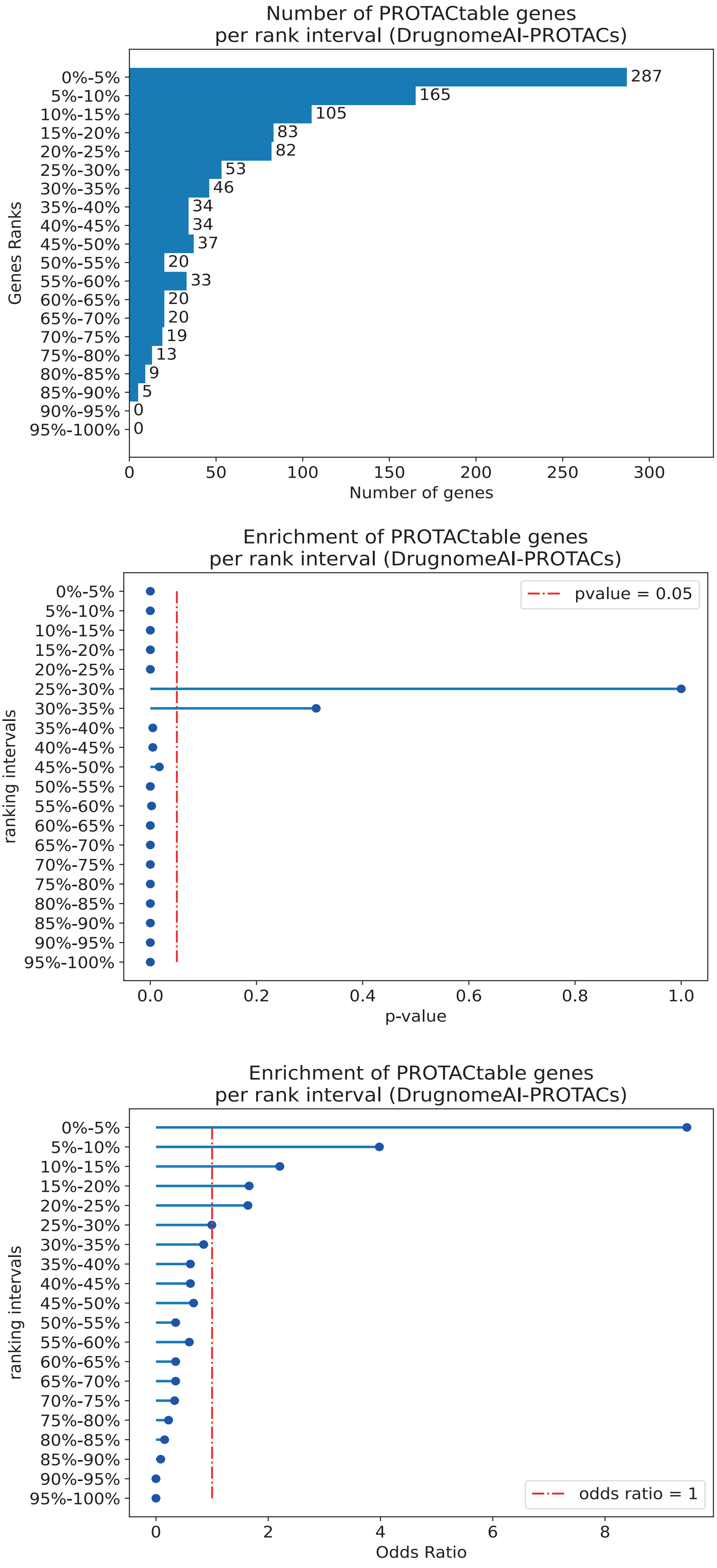

Supplementary Fig. 17 Enrichment of 1,067 genes from Schneider et al per rank interval by the DrugnomeAI PROTACs model. 0%-5% indicates genes ranked in the top 5% while 95%-10-0% indicates the lowest ranked genes.

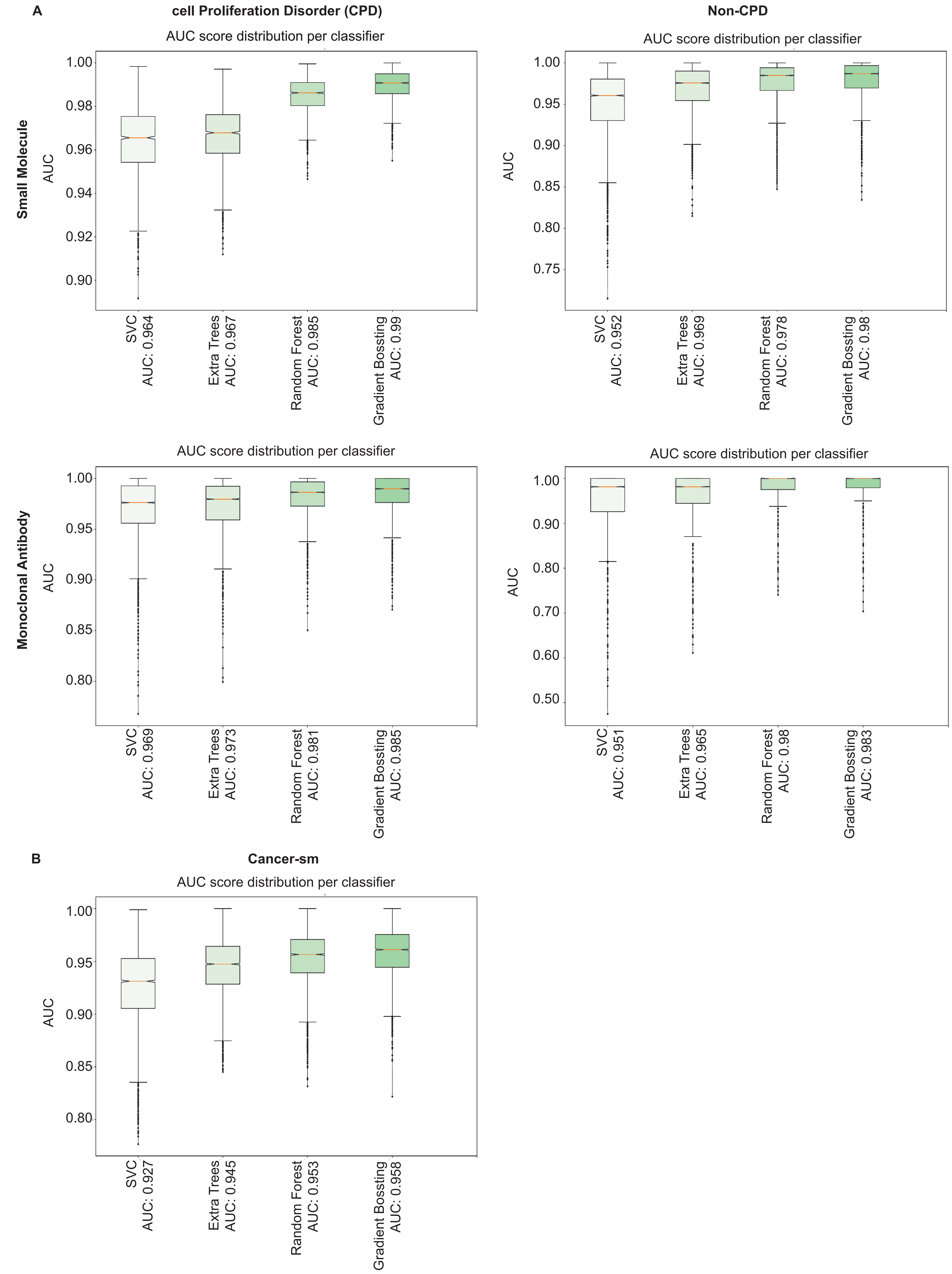

Supplementary Fig. 18 DrugnomeAI models performance for (A) oncology and non-oncology models for small molecules and antibody, and (B) cancer-sm model. The y-asis corresponds to AUC scores, and the x-axis corresponds to a classifier (SVC, Extra Trees, Rrandom Forest, and Gradient Boosting).

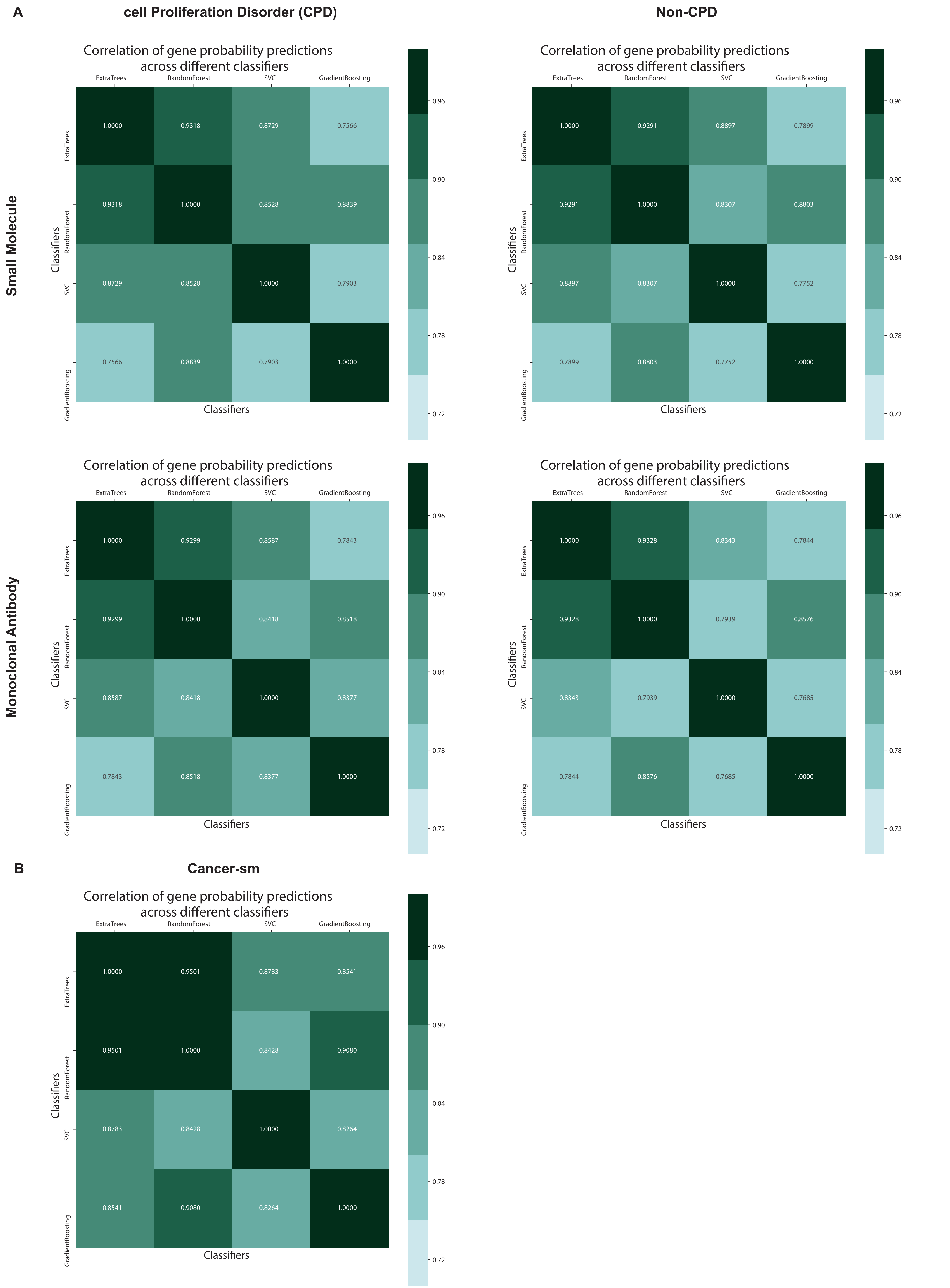

Supplementary Fig. 19 Correlations between predictions of four classifiers (Gradient Boosting, SVC, Random Forest, Extra Trees) for (A) oncology and non-oncology models for small molecules and antibody, and (B) cancer-sm model.

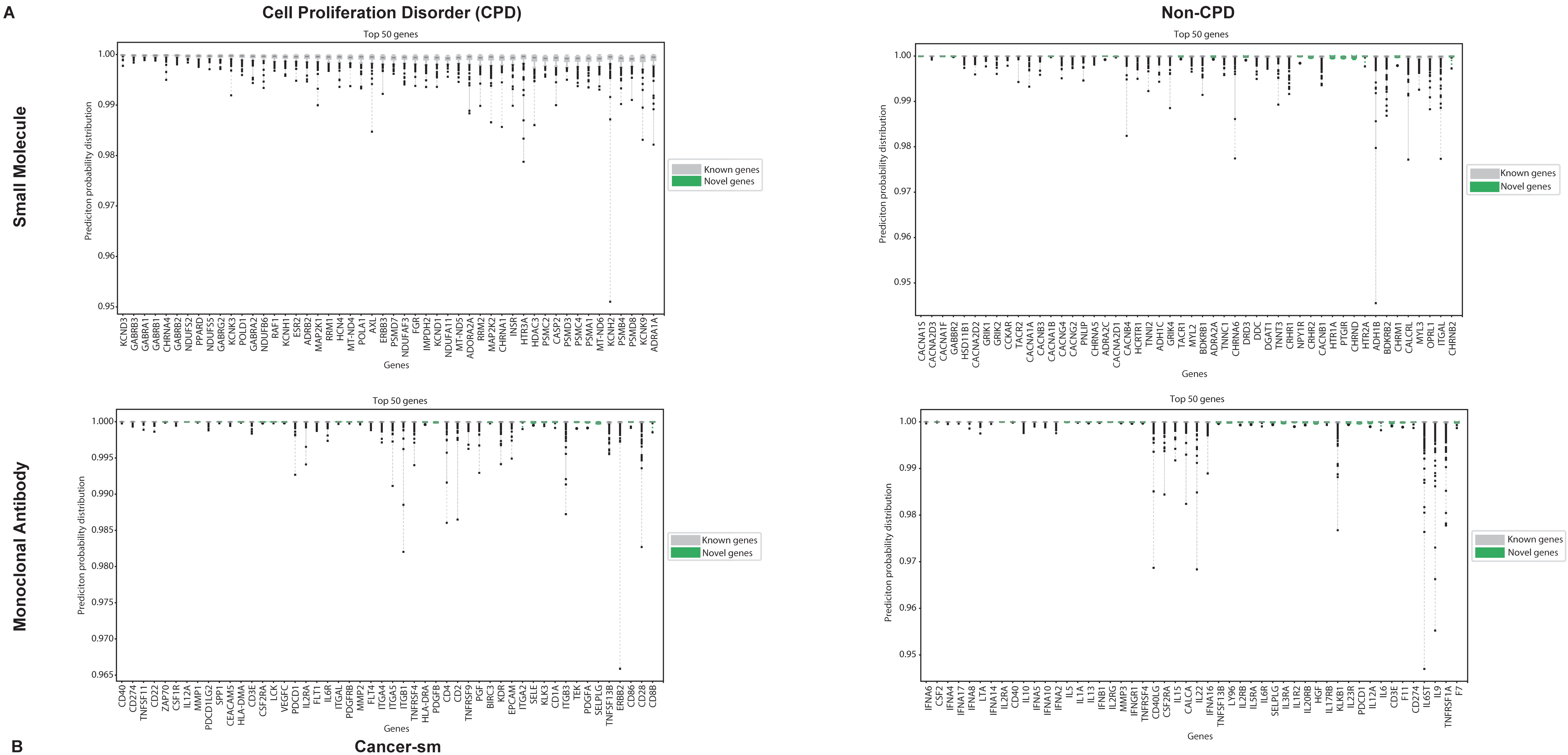

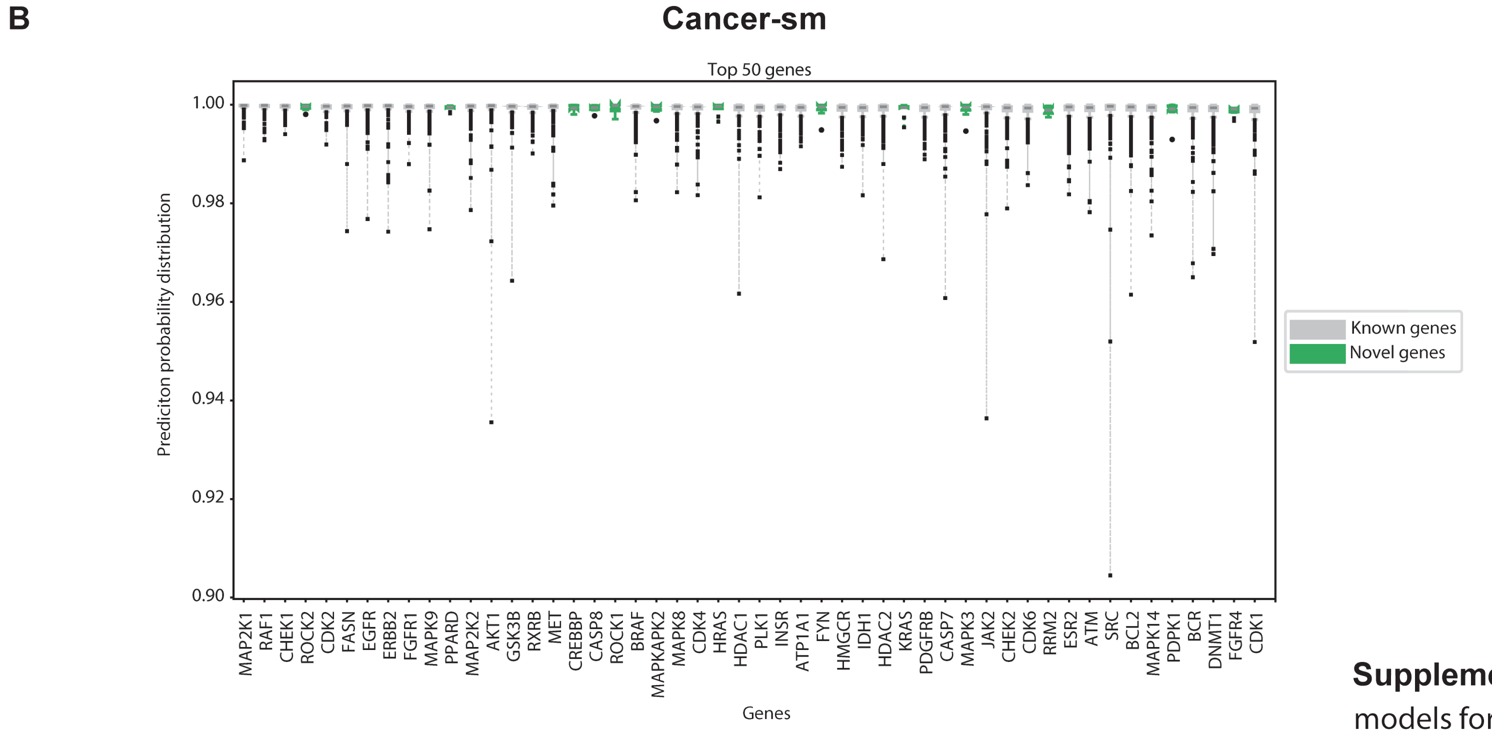

Supplementary Fig. 20 Novel genes in top 5% ranked genes by gradient boosting models for (A) oncology and non-oncology models for small molecules and antibody, and (B) cancer-sm model.

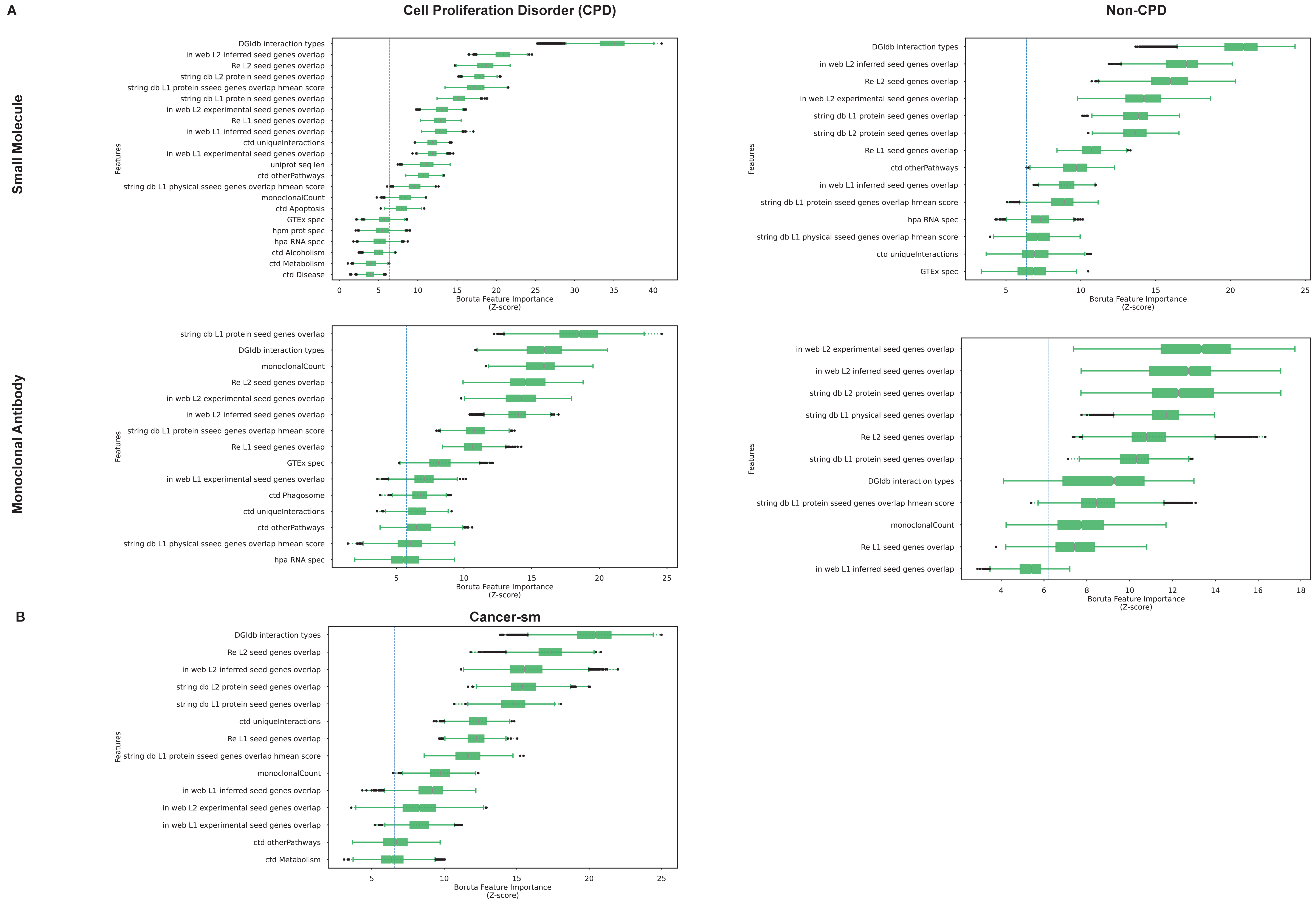

Supplementary Fig. 21 Confirmed features by Boruta analysis for (A) oncology and non-oncology models for small molecules and antibody, and (B) cancer-sm model.

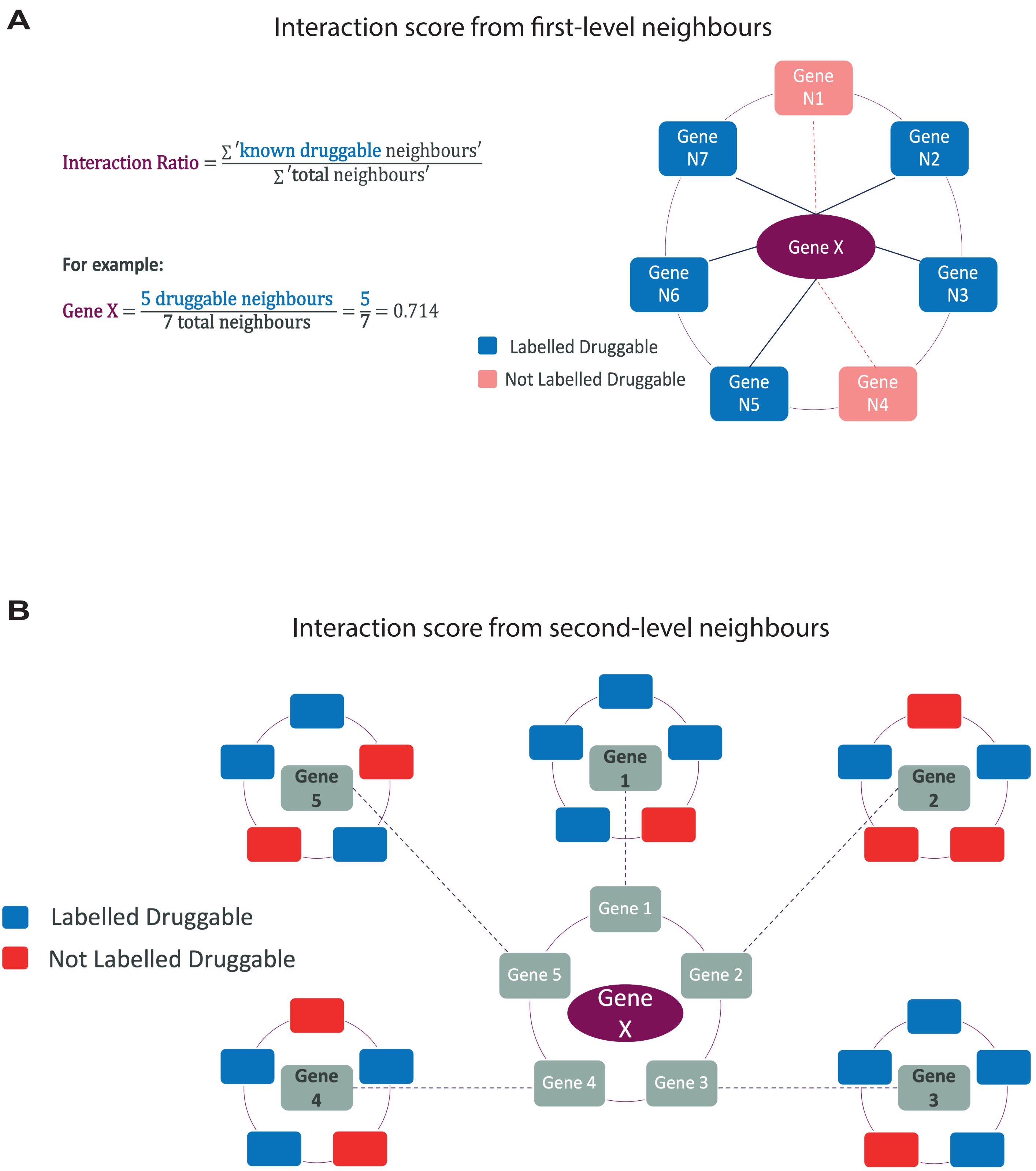

Supplementary Fig. 22 Feature engineering of protein-protein interation networks. (A) Interaction score from first level neighbours is calculated by dividing the number of druggable neighbours (blue) by the total number of neighbours. (B) Interaction score from second-level neighbours is calculated by dividing the number of druggable second neighbours (blue) by total number of second neighbours.

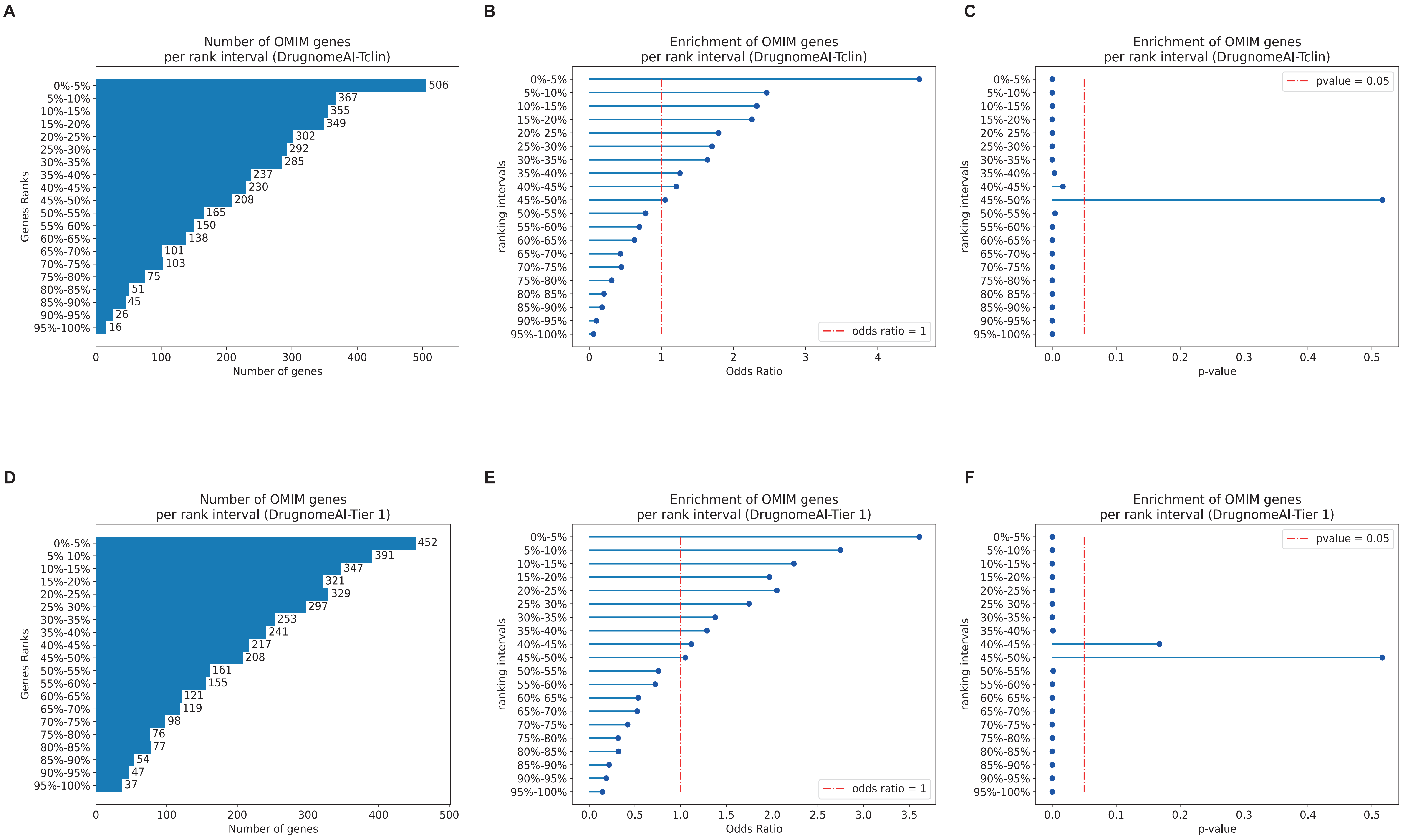

Supplementary Fig. 23 Enrichment of top 5% ranked genes by DrugnomeAI among genes with OMIM associations. 0%-5% indicates genes ranked in the top 5% while 95%-10-0% indicates the lowest ranked genes

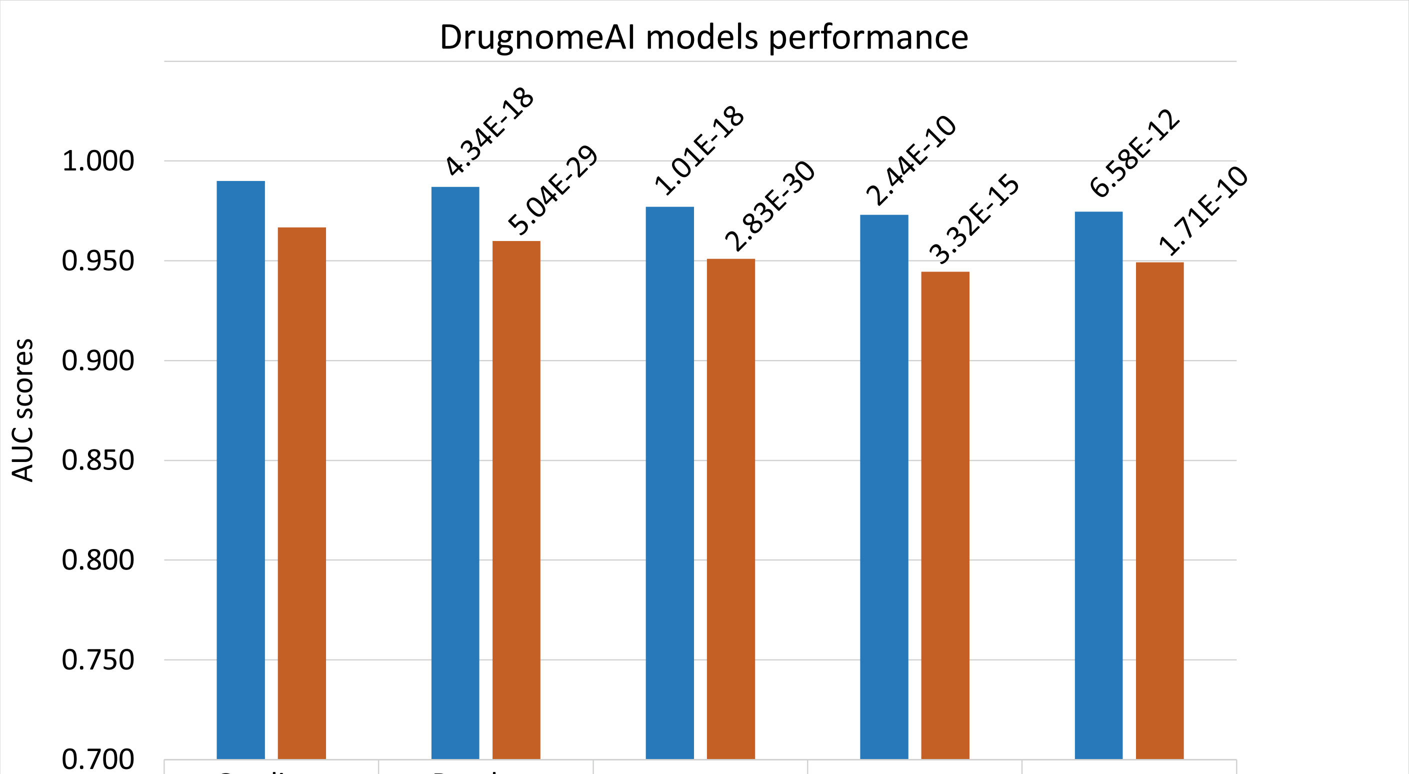

	Gradient Boosting	Random Forest	Extra Tree	SVC	DNN
Tclin	0.990	0.987	0.977	0.973	0.975
Tier1	0.967	0.960	0.951	0.945	0.949

Supplementary Fig. 24 Comparision of five models' AUC scores. The bottom table shows the AUC scores of each model on Tclin and Tier 1 datasets. DeLong p-values are shown on top of bars to denote the statistical significance of gradient boosting performance against the other four models.

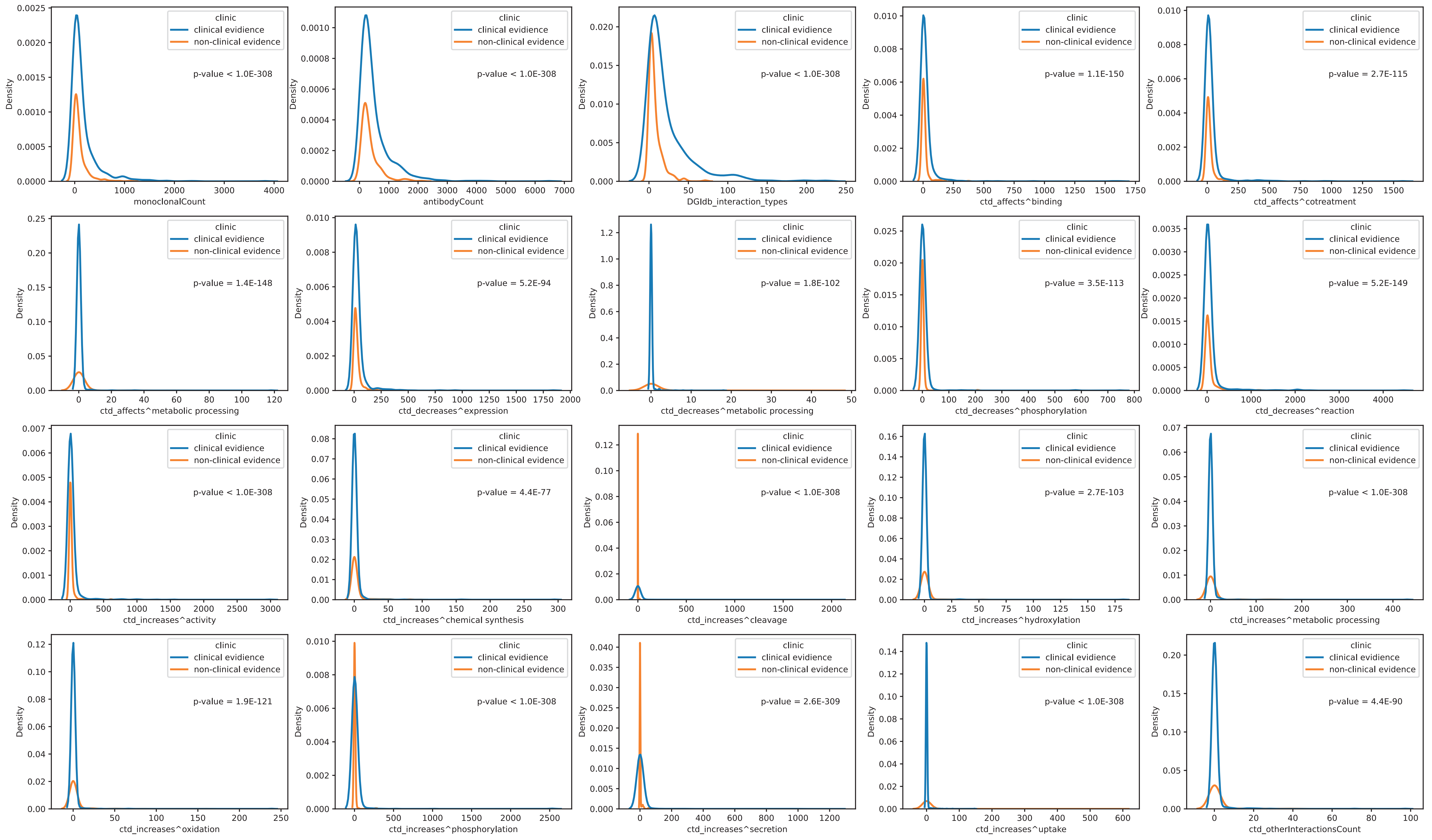

Supplementary Fig. 25 Top 20 features distinguishing top 5% DrugnomeAI-Tclin genes that were selected for clinical development (753 genes) from genes that were not (239 genes). Features are ranked using Chi2 test, and Chi2 test p-values are shown for each feature. Kernel density etimate (KDE) plots demonstrate the distribution of features in each group of genes.

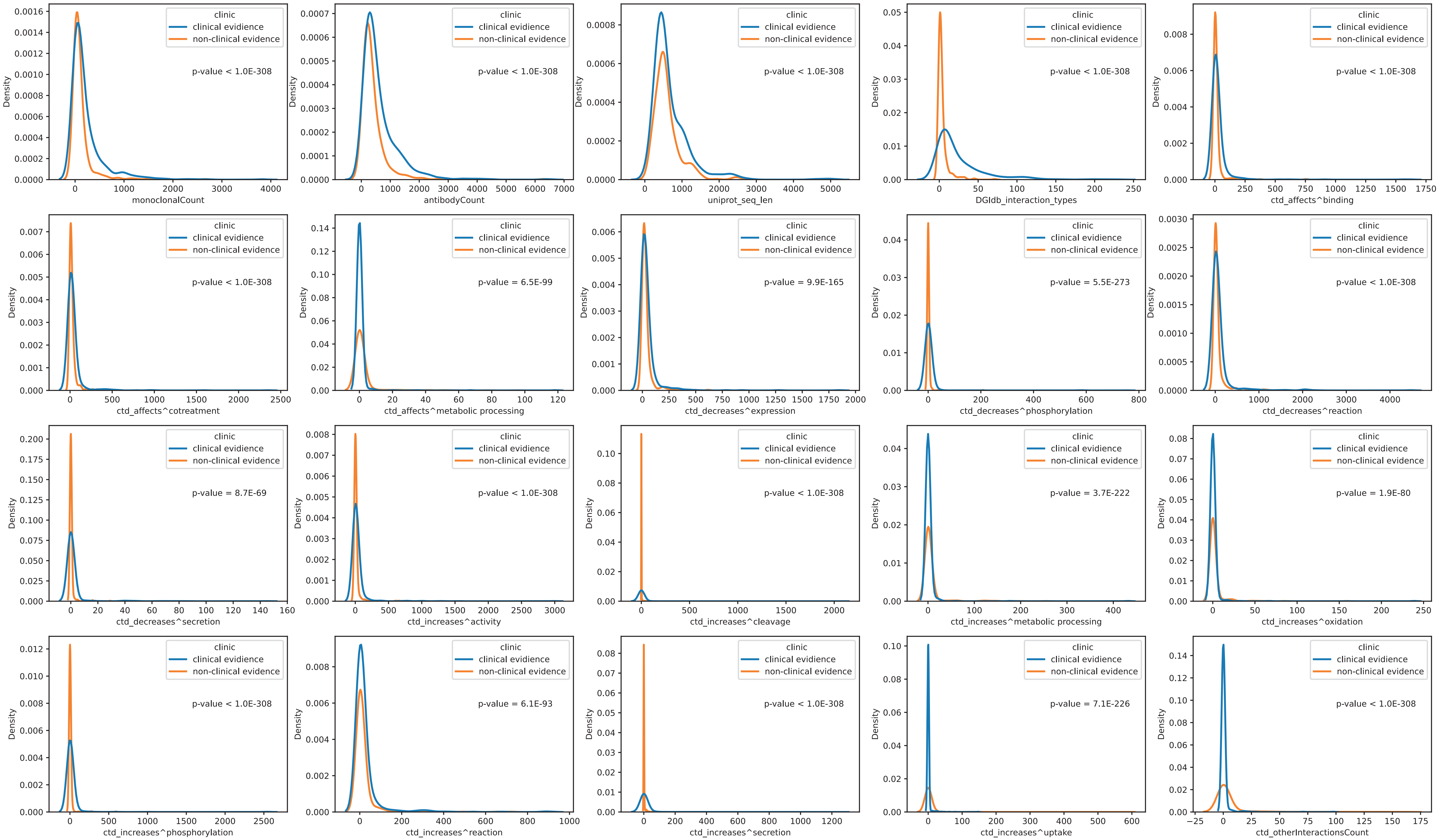

Supplementary Fig. 26 Top 20 features distinguishing top 5% DrugnomeAI-Tier1 genes that were selected for clinical development (605 genes) from genes that were not (387 genes). Features are ranked using Chi2 test, and Chi2 test p-values are shown for each feature. Kernel density etimate (KDE) plots demonstrate the distribution of features in each group of genes.

A

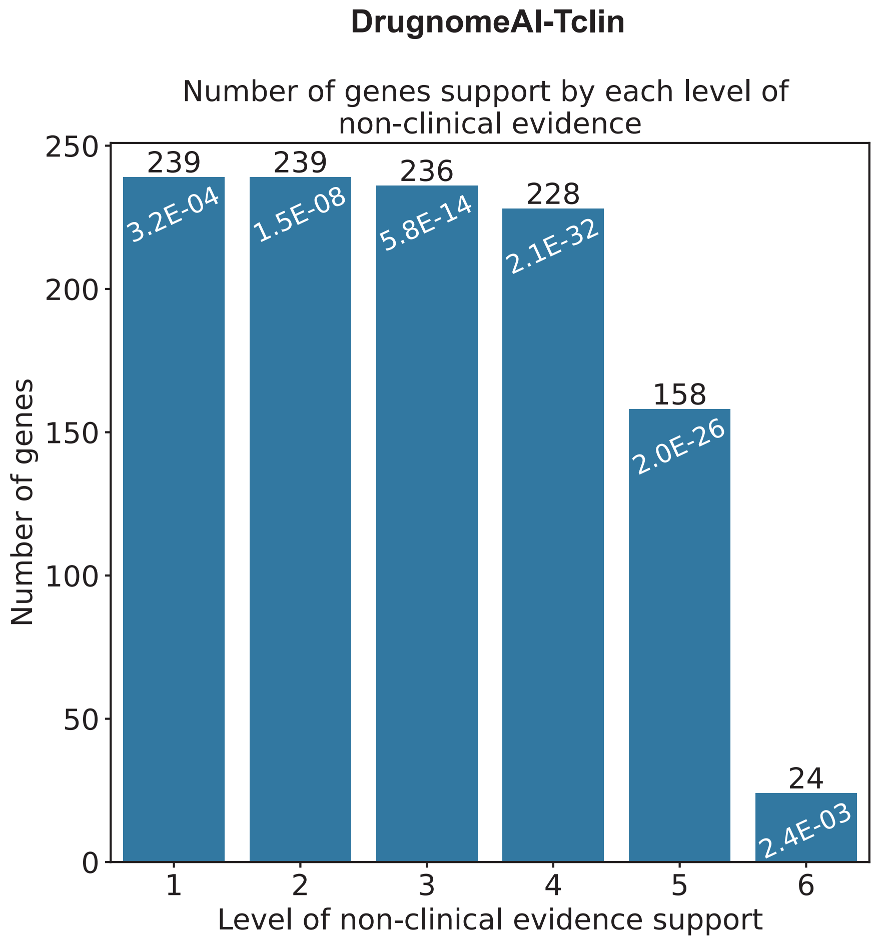

B

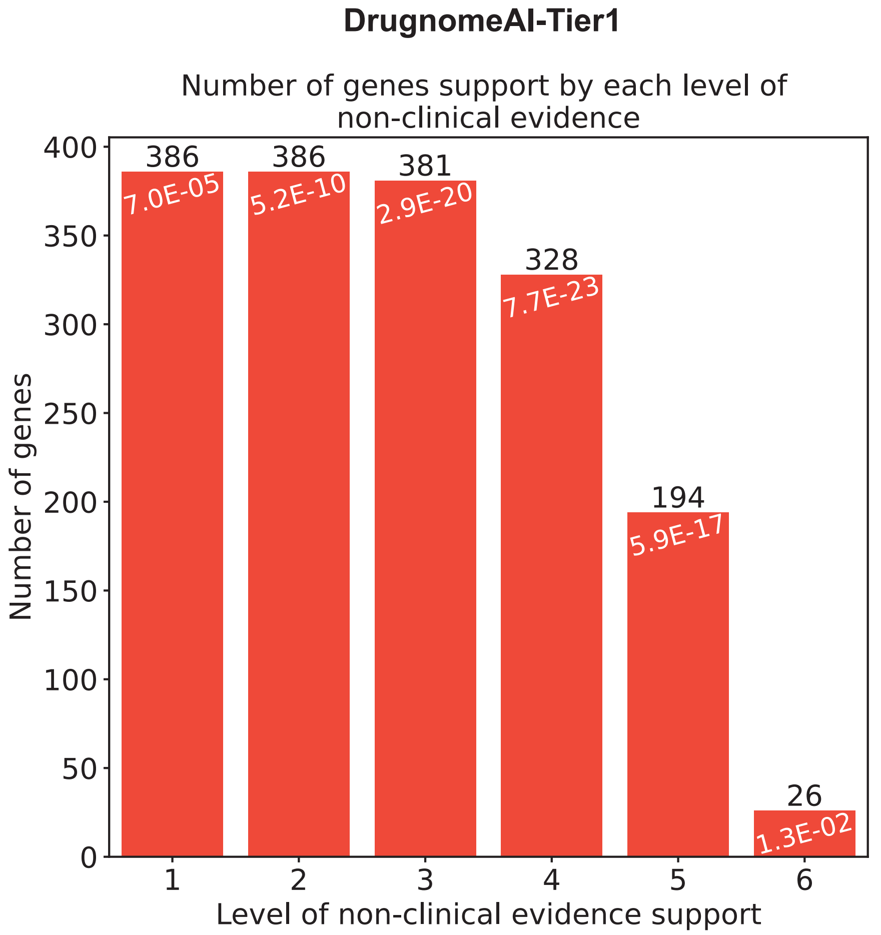

Supplementary Fig. 27 Top 5% DrugnomeAI genes that were not selected for clinical development from (A) DrugnomeAI-Tcln (239 genes) and (B) DrugnomeAI-Tier1 (387 genes). The figure demonstrates the level of support from non-clinical evidnece (e.g., 24 genes from DrgunomeAI-Tclin are supported by 6 types of non-clinical evidence while 239 genes are supported by at least two types of non-clinical evidence).

A

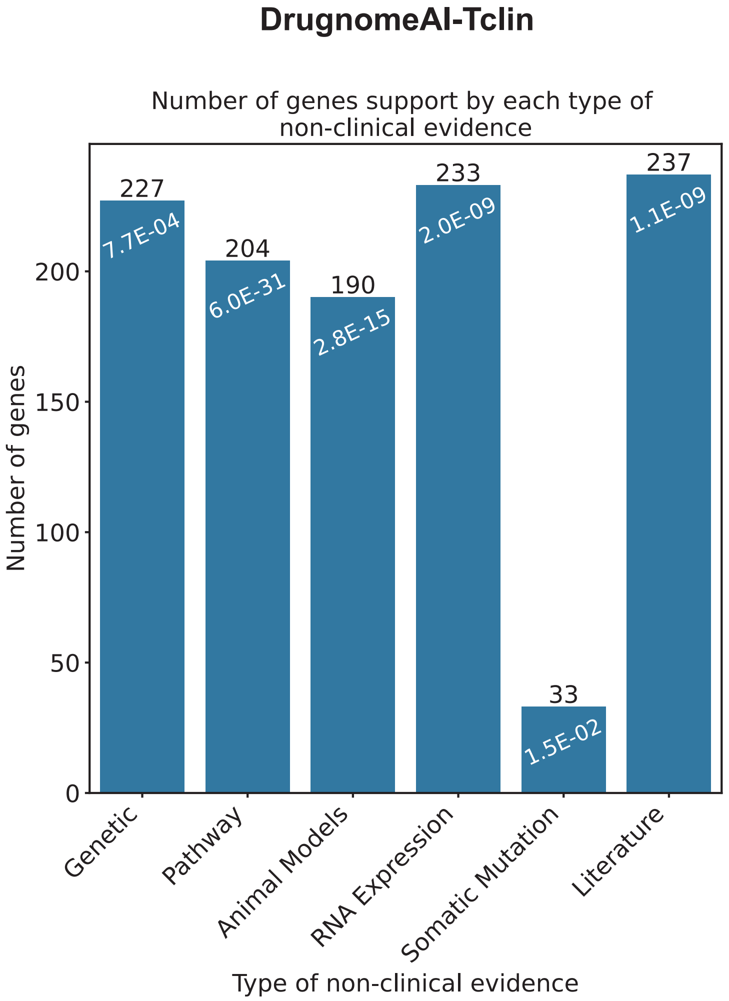

B

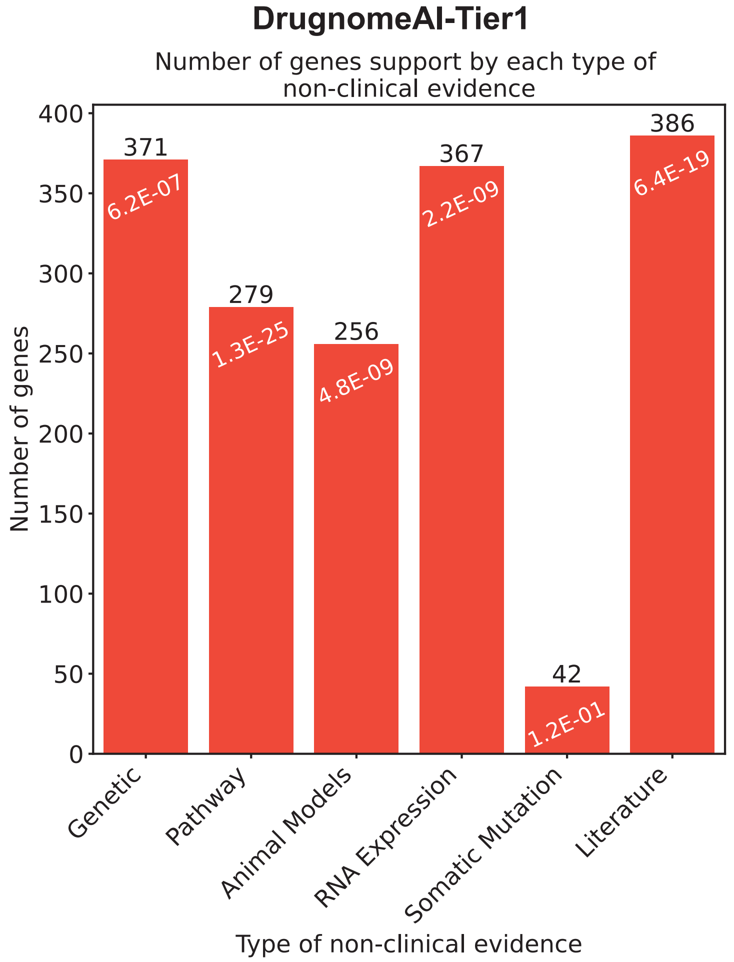

Supplementary Fig. 28 Top 5% DrugnomeAI genes that were not selected for clinical development from (A) DrugnomeAI-Tcln (239 genes) and (B) DrugnomeAI-Tier1 (387 genes). The figure demonstrates the number of genes supported by each type of non-clinical evidnece.
