# Supplementary material for: DrugnomeAI is an ensemble machine-learning framework for predicting druggability of candidate drug targets
Source: Commun Biol. 2022 Nov 24;5:1291. doi: 10.1038/s42003-022-04245-4 (PMC9700683; doi:10.1038/s42003-022-04245-4)
Supplement: Supplementary file 3 — Description of Additional Supplementary Files [file 42003_2022_4245_MOESM3_ESM.pdf]

Description of Additional Supplementary Files

File name: Supplementary Data 1
Description: Lists of top ranked genes.

File name: Supplementary Data 2
Description: Overlap between top ranked genes by DrugnomeAI models.

File name: Supplementary Data 3
Description: Clinical evidence of top ranked DrugnomeAI genes.

File name: Supplementary Data 4
Description: Non-clinical evidence of top ranked DrugnomeAI genes.

File name: Supplementary Data 5
Description: Features descriptions.

File name: Supplementary Data 6
Description: Erichment with OMIM analysis.

File name: Supplementary Data 7
Description: Enrichment of DrugnomeAI models overlap with validation sets compared to published tools based on Fisher's exact test.

File name: Supplementary Data 8
Description: Comparison with published tools.

File name: Supplementary Data 9
Description: List of data sources of features and list of final DrugnomeAI features.
