# Supplementary material for: DrugnomeAI is an ensemble machine-learning framework for predicting druggability of candidate drug targets
Source: Commun Biol. 2022 Nov 24;5:1291. doi: 10.1038/s42003-022-04245-4 (PMC9700683; doi:10.1038/s42003-022-04245-4)
Supplement: Supplementary file 13 — Reporting Summary [file 42003_2022_4245_MOESM13_ESM.pdf]

nature portfolio

Corresponding author(s): Dimitrios Vitsios

Last updated by author(s): May 3, 2022

Reporting Summary

Nature Portfolio wishes to improve the reproducibility of the work that we publish. This form provides structure for consistency and transparency in reporting. For further information on Nature Portfolio policies, see our Editorial Policies and the Editorial Policy Checklist.

Statistics

For all statistical analyses, confirm that the following items are present in the figure legend, table legend, main text, or Methods section.

n/a	Confirmed	
☐	☒	The exact sample size (*n*) for each experimental group/condition, given as a discrete number and unit of measurement
☐	☒	A statement on whether measurements were taken from distinct samples or whether the same sample was measured repeatedly
☐	☒	The statistical test(s) used AND whether they are one- or two-sided<br>*Only common tests should be described solely by name; describe more complex techniques in the Methods section.*
☒	☐	A description of all covariates tested
☐	☒	A description of any assumptions or corrections, such as tests of normality and adjustment for multiple comparisons
☐	☒	A full description of the statistical parameters including central tendency (e.g. means) or other basic estimates (e.g. regression coefficient) AND variation (e.g. standard deviation) or associated estimates of uncertainty (e.g. confidence intervals)
☐	☒	For null hypothesis testing, the test statistic (e.g. *F*, *t*, *r*) with confidence intervals, effect sizes, degrees of freedom and *P* value noted<br>*Give P values as exact values whenever suitable.*
☒	☐	For Bayesian analysis, information on the choice of priors and Markov chain Monte Carlo settings
☒	☐	For hierarchical and complex designs, identification of the appropriate level for tests and full reporting of outcomes
☒	☐	Estimates of effect sizes (e.g. Cohen's *d*, Pearson's *r*), indicating how they were calculated

Our web collection on statistics for biologists contains articles on many of the points above.

Software and code

Policy information about availability of computer code

Data collection	All code for data collection is publicly available on GitHub:<br>https://github.com/astrazeneca-cgr-publications/DrugnomeAI-release<br>and more particularly in the following modules:<br>https://github.com/astrazeneca-cgr-publications/DrugnomeAI-release/tree/master/drugnome_ai/modules/pre_processing/data_compilation<br>https://github.com/astrazeneca-cgr-publications/DrugnomeAI-release/tree/master/drugnome_ai/data
Data analysis	The DrugnomeAI package and code for training the models, reproducing all validation analyses (along with the training/validation datasets) and instructions for installing and running the software are available in the GitHub repository: https://github.com/astrazeneca-cgr-publications/DrugnomeAI-release.

For manuscripts utilizing custom algorithms or software that are central to the research but not yet described in published literature, software must be made available to editors and reviewers. We strongly encourage code deposition in a community repository (e.g. GitHub). See the Nature Portfolio guidelines for submitting code & software for further information.

Data

Policy information about availability of data

All manuscripts must include a data availability statement. This statement should provide the following information, where applicable:

- Accession codes, unique identifiers, or web links for publicly available datasets
- A description of any restrictions on data availability
- For clinical datasets or third party data, please ensure that the statement adheres to our policy

Labelled gene lists from Pharos are available at: https://github.com/astrazeneca-cgr-publications/DrugnomeAI-release/drugnome_ai/data/PHAROS/

pharos_GF_wINDEX.csv.
Triage gene lists are available at: https://github.com/astrazeneca-cgr-publications/DrugnomeAI-release/blob/master/drugnome_ai/data/labels/gene_druggable_labels.csv.
Labeled gene lists for training specialised models are available at: https://github.com/astrazeneca-cgr-publications/DrugnomeAI-release/misc/gene_lists.
The data used in this study is obtained from the following sources: PHAROS (http://juniper.health.unm.edu/tcrd), InWeb (https://www.intomics.com/inbio/map.html#downloads), StringDB (https://string-db.org), Reactome (https://reactome.org), DGIdb (www.dgidb.org), CTDbase (http://ctdbase.org), InterPro (https://www.ebi.ac.uk/interpro), OMIM (https://www.omim.org), Open Targets Platform (https://platform.opentargets.org/downloads), and CTRP (https://portals.broadinstitute.org/ctrp.v2.1/?page=#ctd2Target).
Additionally, we used datasets from the following publications: Finan et al. (https://www.ncbi.nlm.nih.gov/pmc/articles/PMC6321762/#SMtitle), King et al. (https://journals.plos.org/plosgenetics/article?id=10.1371/journal.pgen.1008489#sec018), Wang et al. (https://www.nature.com/articles/s41586-021-03855-y#data-availability), and Schneider et al. (https://www.nature.com/articles/s41573-021-00245-x#Sec10), and Costa et al. (https://bmcgenomics.biomedcentral.com/articles/10.1186/1471-2164-11-S5-S9#additional-information).

The extracted DrugnomeAI predictions across all models and all protein-coding genes are available at the DrugnomeAI web-app (Browse tab): https://astrazeneca-cgr-publications.github.io/DrugnomeAI

Field-specific reporting

Please select the one below that is the best fit for your research. If you are not sure, read the appropriate sections before making your selection.

☒ Life sciences ☐ Behavioural & social sciences ☐ Ecological, evolutionary & environmental sciences

For a reference copy of the document with all sections, see nature.com/documents/nr-reporting-summary-flat.pdf

Life sciences study design

All studies must disclose on these points even when the disclosure is negative.

Sample size	In the context of this work, sample sizes refer to the number of labeled genes considered as the positive (known druggable) gene set in each of the trained DrugnomeAI models. These are mentioned in Table 1 (Methods) for the different labeled sets derived from Pharos and the Tier/Triage resources, specifically: Tclin (n=610), Tchem (n=1,592), Tbio (n=11,316), Tier 1 (n=1,411), Tier 2 (n=658), Tier 3A (n=845), Tier 3B (n=1,437). Multiple balanced test sets are constructed within the stochastic semi-supervised learning framework of DrugnomeAI (with a 3:2 ratio of negative to positive data points in each balanced dataset), covering the entire exome space (n=19,846) hundreds of times.
Data exclusions	No data were excluded.
Replication	Replication has been performed in the form of cross-validation across multiple stochastic iterations, allowing for different random balanced subsets to be used for training and then validation on thousands of hold-out datasets.
Randomization	Randomization has been performed in the form of randomly partitioning the entire gene space into balanced datasets and then applying cross-validation on random splits of each of those balanced datasets.
Blinding	n/a

Reporting for specific materials, systems and methods

We require information from authors about some types of materials, experimental systems and methods used in many studies. Here, indicate whether each material, system or method listed is relevant to your study. If you are not sure if a list item applies to your research, read the appropriate section before selecting a response.

Materials & experimental systems

n/a	Involved in the study	
☒	☐	Antibodies
☒	☐	Eukaryotic cell lines
☒	☐	Palaeontology and archaeology
☒	☐	Animals and other organisms
☒	☐	Human research participants
☒	☐	Clinical data
☒	☐	Dual use research of concern

Methods

n/a	Involved in the study	
☒	☐	ChIP-seq
☒	☐	Flow cytometry
☒	☐	MRI-based neuroimaging
